# General and Mild Method for the Synthesis of Polythioesters from Lactone Feedstocks

**DOI:** 10.1021/acsmacrolett.4c00556

**Published:** 2024-10-08

**Authors:** McKinley K. Paul, Matthew C. Raeside, Will R. Gutekunst

**Affiliations:** School of Chemistry and Biochemistry, Georgia Institute of Technology, 901 Atlantic Drive NW, Atlanta, Georgia 30332, United States

## Abstract

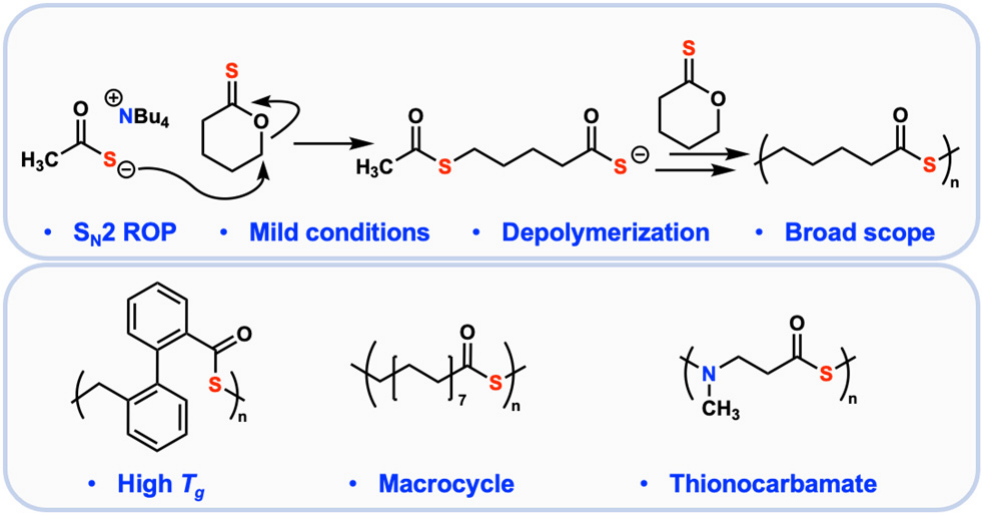

Polythioesters are attracting increasing interest in applications requiring degradability or recyclability. However, few general methods exist for the synthesis of these polymers. This report presents a fast and versatile method for synthesizing polythioesters from readily available lactone feedstocks. The two-step process begins with the thionation of lactones to thionolactones, followed by the ring-opening polymerization of the thionolactones to polythioesters. Unlike previous methods that rely on harsh reagents to accomplish this transformation, we demonstrate that the mild tetrabutylammonium thioacetate is a competent initiator for polymerization. This method exhibits broad applicability, as demonstrated by the successful polymerizations of an unstrained 17-membered macrocycle and an N-substituted cyclic thionocarbamate. Furthermore, the generality of this scheme enables the synthesis of polythioesters with highly tunable properties, as demonstrated here by the synthesis of a set of polymers with glass transition temperatures spanning 180 °C. Finally, the polythioesters are efficiently depolymerized into the corresponding thiolactones.

Polythioesters have garnered significant attention in the literature as a promising class of materials with applications in the development of degradable and recyclable polymers.^1−21^ Most commonly, polythioesters are synthesized through ring-opening polymerization (ROP) of thiolactones, as demonstrated by Kiesewetter and colleagues.^16^ However, this conventional approach suffers from several drawbacks. For example, there are a limited number of commercial reagents that can be transformed into thiolactone monomers in a reasonable number of steps. Additionally, the traditional ROP process demands that the monomer possess sufficient ring strain to drive polymerization.^22^ This constraint on monomer design limits the scope of accessible polythioesters. Thus, the chemical diversity in this class of polymers is limited by the few, nongeneral methods for synthesizing thiolactone monomers.

Preparation of polythioesters from lactone starting materials is an ideal solution to this problem, as lactones could provide inexpensive, structurally diverse, and potentially bioderived feedstocks. A promising two-step pathway to accomplish this transformation is the thionation of lactones to thionolactones, followed by a polymerization step, during which the C=S thionolactone monomers isomerize to produce C=O polythioesters. In contrast to the 1,2-addition–elimination mechanism conventionally employed in ROP of thionolactones,^23,24^ polymerization of thionolactones to polythioesters occurs via S_N_2 reaction at the C–O carbon in the lactone ring, expelling a thioacetate chain end for propagation (Figure 1d).^2,25^

**Figure 1:**
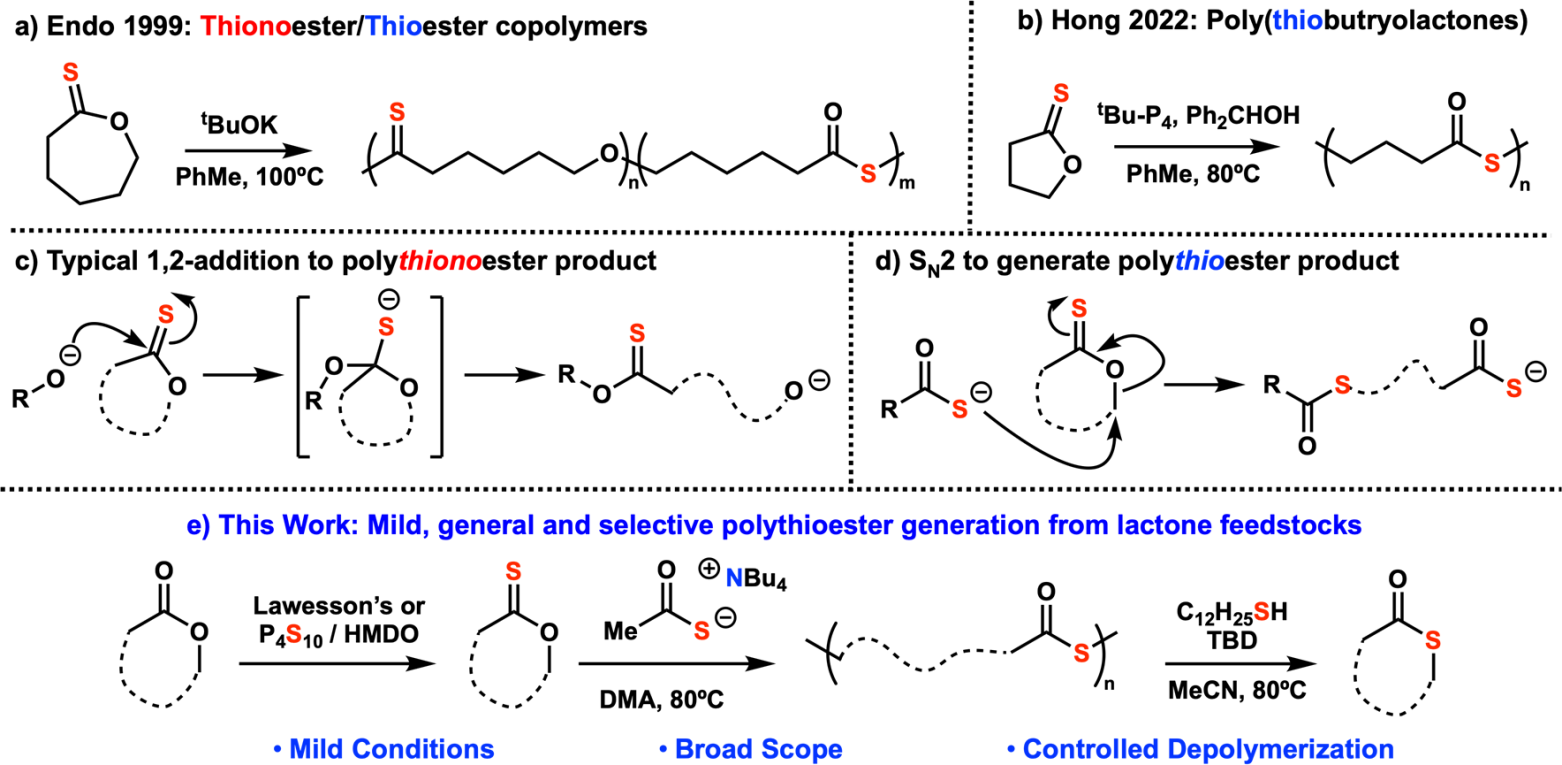
Overview of the previous work. (a) Initial work by Endo demonstrating conversion of thionolactones to polythioesters in a polythionoester/polythioester copolymer.^26^ (b) Yuan et al.’s recent work showing full selectivity to polythioester products with phosphazene superbase initiator system.^25^ (c) Typical 1,2-addition–elimination mechanism expected in ROP of thionolactones which generates polythionoesters. (d) S_N_2 of thionolactones to generate polythioesters. (e) This work: mild, general, and selective conversion of thionolactones to polythioester followed by depolymerization to thiolactone small molecules.

Endo and co-workers first demonstrated the viability of a thionolactone to polythioester transformation using harsh initiators (such as organolithiums or potassium *tert*-butoxide). Unfortunately, these conditions resulted in low selectivity of polythioester versus polythionoester products (Figure 1a).^26^ However, Endo’s later investigations found that treatment of thionolactones with cationic initiators results exclusively in polythioester products.^27,28^ More recently, Hong and co-workers demonstrated that treating thionolactones with phosphazene superbases and diphenylmethanol (or highly electrophilic oxonium salts) achieved complete selectivity to polythioester products with high molecular weights and fair dispersities (Figure 1b).^25,29^ Extensive computational studies and end-group analysis via ESI-MS performed by Hong and co-workers showed that polymerization under anionic conditions occurs via propagating thiocarboxylate chain ends. However, only polymerization of five-membered substrates were demonstrated in this recent work, and thus the generality of these transformations was unknown before the current study.

Inspired by these findings, we hypothesized that employing a milder initiator, chemically akin to the propagating thioacetate chain end, would facilitate the same transformation under milder conditions (Figure 1e). To test this hypothesis, 100 equiv of thionovalerolactone **1** was treated with 1 equiv of thioacetic acid and 1 equiv of 1,8-diazabicyclo[5.4.0]undec-7-ene (DBU) in dimethylformamide (DMF) at 5 M. The reaction was heated at 80 °C for 1 h which yielded a sole polymer product with 50% conversion. ^13^C NMR of the precipitated polymer indicated that the material was completely the polythioester product, as confirmed by comparison to an independently prepared sample of the polythionoester (Figures S3–S6). Prolonging reaction time to 3 h increased conversion to 79%. Interestingly, gas phase density functional theory calculations at the M06-2x/6-311++G** level of theory indicate that the electrophilic orbital involved in the S_N_2-ROP process is not LUMO but LUMO+1 of the thionolactones (Figures S52 and S53). While the high molecular weight polymer products of the initial test were selectively polythioesters, the crude ^1^H NMR of our test reaction showed small molecule byproducts around 4.0 ppm, similar to those which Hong and co-workers identified as side products resulting from thionoenolate chemistry.^25^ Due to DMF’s dissociation to dimethylamine and carbon monoxide at elevated temperatures,^30^ it was hypothesized that switching solvent to dimethylacetamide (DMA) could reduce the thionoenolate-related byproducts. In line with this thought, performing the same reaction in DMA produced fewer byproducts at around 4.0 ppm (Figure S7).

Encouraged by these preliminary results, we further investigated the effect of solvent and concentration on the reaction. Solvents with various characteristics such as polar, nonpolar, hydrogen bond acceptors, and hydrogen bond donors were screened (Tables S2 and S3). However, DMA showed the best performance of any solvent investigated. Next, different initiator systems were evaluated (Table 1). As a control experiment, thionovalerolactone **1** was subjected to DBU alone (Table 1, entry 2). The observed polymerization in this control indicates either that DBU itself is able to directly perform the initiating S_N_2 to give a zwitterionic ring-opened species or that DBU is able to deprotonate a monomer, generating a thionoenolate species capable of nucleophilic initiation as suggested by Hong and co-workers.^25^ To explore the effect of different nucleophiles, benzyl mercaptan and diphenylmethanol were screened in combination with DBU (Table 1, entries 3 and 4). While both systems were found to initiate polymerization, they showed inferior performance to that of the thioacetic acid and DBU combination. As hydrogen bonding was invoked in the exploration of the phosphazene/diphenylmethanol initiation system,^25^ hydrogen bond donors were added to the DBU and thioacetic acid system with the goal of increasing polymerization control. Hexafluoroisopropanol (HFIP) and diphenylmethanol were selected for this purpose (Table 1, entries 5 and 6). Both H-bond donors reduced the conversion that took place in 3 h, and neither donor had a positive impact on the dispersity of polymerization. After the thioacetate anion was established as the best performing nucleophile, various bases were investigated.

**Table 1:**
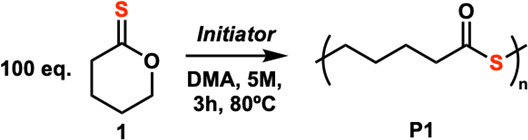

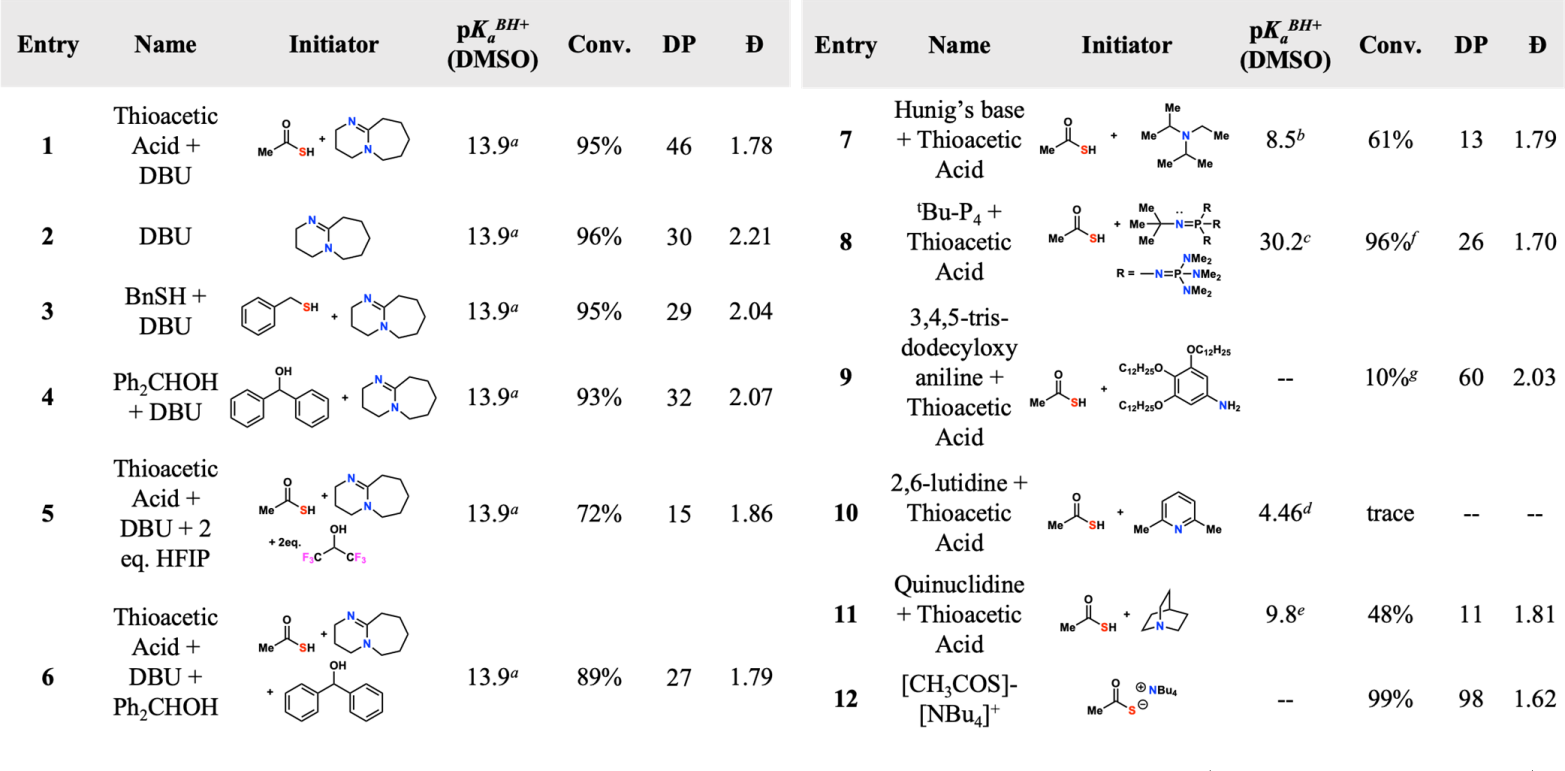
Results of the Initiator System Screening

Conditions: 100 equiv of thionovalerolactone **1**, 1 equiv of initiator, DMA, 5 M, 3 h, 80 °C, quenched with excess TFA. (a) Value from ref (31). (b) Value from ref (34). (c) Value from ref (33). (d) Value from ref (35). (e) Value from ref (36). (f) Reaction in toluene, 3 M, 0.5 h. (g) Reaction in toluene, 5 M, 3 h.

Hypothesizing that a sterically bulky countercation could hinder chain transfer and backbiting reactions (Figure S10), several bulky bases were screened in combination with thioacetic acid including Hünig’s base, ^t^Bu-P_4_, an electron-rich aniline, and 2,6-lutidine (Table 1, entries 7–10). Unfortunately, none of these bases showed significantly superior performance compared with DBU and thioacetic acid. ^t^Bu-P_4_, however, did show a slightly decreased dispersity compared to DBU. Along another line of investigation, if the polymerization observed in the control experiment of treating monomer with DBU alone (Table 1, entry 2) was evidence of DBU acting directly as a nucleophilic initiator to generate a zwitterionic chain end, it would stand to reason that using a more nucleophilic amine would improve the polymerization performance. For this purpose, highly nucleophilic quinuclidine was screened (Table 1, entry 11). However, the quinuclidine and thioacetic acid combination showed much lower conversion than the DBU and thioacetic acid combination. This result, combined with the 10 orders of magnitude *K*_a_ difference between thioacetic acid and DBU (p*K*_a_^BH*+*^_H_2_O_ = 13.5 for DBU compared to p*K*_a_^BH^_H_2_O_ = 3.4 for thioacetic acid),^31,32^ indicates that DBU is acting as a Brønsted base rather than a nucleophile in the thioacetic acid/DBU initiation system. This hypothesis is bolstered by the high conversion and low dispersity resulting from use of the phosphazene super base (p*K*_a_^BH*+*^_DMSO_ = 30.2 for the phosphazene compared to p*K*_a_^BH*+*^_DMSO_ = 13.9 for DBU).^31,33^ Furthermore, weaker bases, such as Hünig’s base and 2,6-lutidine, both showed lower conversions than DBU, with 2,6-lutidine showing no conversion at all. From these results, it was concluded that stronger bases likely result in higher conversions and lower dispersities due to their production of a higher concentration of the true nucleophilic initiator, the thioacetate anion. To test this theory, the acid–base equilibrium was removed from the equation by testing initiation via addition of the organic soluble tetrabutylammonium thioacetate salt (Table 1, entry 12). Satisfyingly, the thioacetate salt showed the best performance of any initiator screened, matching the conversion of the phosphazene base and thioacetic acid system but with slightly decreased dispersity. Kinetic experiments were also performed (Figure S54), showing first-order behavior for this system.

After optimal polymerization conditions were established, the generality of the reaction was explored (Table 2). A variety of thionolactone monomers were prepared from the corresponding lactones by thionation with either P_4_S_10_ or Lawesson’s reagent. The thionolactone monomers were then polymerized with the tetrabutylammonium thioacetate salt in DMA at 5 M/80 °C at a target degree of polymerization (DP) of 200. The homologous series of five (**2**), six (**1**), and seven membered (**3**) unsubstituted thionolactones were initially polymerized. All were polymerized to high conversions with moderate dispersities, demonstrating the generality of the reaction with respect to the ring size. The amount of small molecule thiolactone byproducts, arising from backbiting during polymerization (Figure S10), was greater in the less strained five membered system compared to the more strained six and seven membered systems. However, the thiolactone-producing backbiting reaction could largely be mitigated by decreasing the reaction time. With these results established, monomer **4** was then polymerized. This polymer, **P4**, was previously unable to be synthesized in a prior report due to the lack of ring strain in the corresponding thiolactone.^22^ The synthesis of **P4** via the method reported here highlights that the primary driving force of polymerization is the energy released upon C=S to C=O isomerization and not the release of ring strain energy as in ring-opening polymerizations of thiolactones. Thus, removing the consideration of ring strain from monomer design via the synthetic pathway reported here further increases the number and diversity of synthetically accessible polythioesters.

**Table 2:**
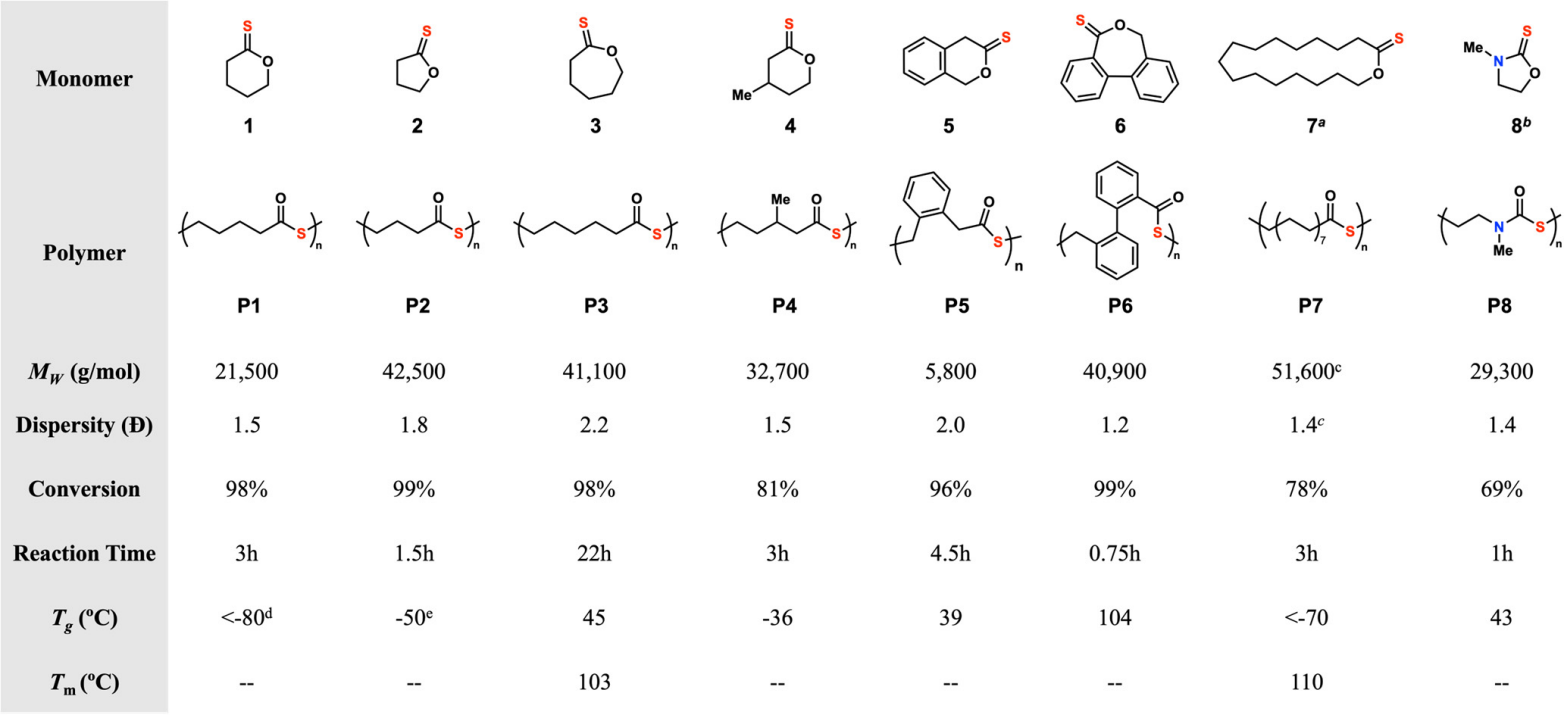
Exploration of the Substrate Scope

Polymerization conditions: 200 equiv of monomer, 1 equiv of CH_3_COS NBu_4_, 5 M DMA, 80 °C, quenched with excess TFA. (a) 200 equiv of monomer, 1 equiv of thioacetic acid, 1 equiv of DBU, DMPU 1 M, 145 °C, 3 h, quenched with excess TFA. (b) 200 equiv of monomer, 1 equiv of CH_3_COS NBu_4_, solvent free, 100 °C, 2 h, quenched with excess TFA. (c) Measured via multiangle laser light scattering (MALLS). (d) Value from ref (22). (e) Value from ref (25).

However, although the above examples demonstrate the broad applicability of this reaction, several limitations were found. The phthalide-derived monomer **S1** (Figures S11 and S12) was not able to be polymerized, and instead only small molecule isomerization of the thionolactone to the corresponding thiolactone was observed. This outcome is hypothesized to arise from a Thorpe–Ingold-like effect,^37,38^ in which the close proximity of the thioacetate and thioester in the ring-opened conformation causes ring closure to become highly favorable. Moreover, monomer **S2**, a six membered thionolactone methylated at the S_N_2 carbon, was unreactive in polymerization. This result underscores a predictable limitation in this polymerization scheme: steric hindrance at the S_N_2 site inhibits the reaction. This result is in line with the previous report, which found that the analogous five membered thionolactone methylated at the S_N_2 site did not polymerize upon treatment with the phosphazene initiator system.^25^

As polythioesters often show glass transition temperatures below room temperature,^22^ several monomers with rigid cyclic groups were targeted with the hopes of achieving glass transition temperatures above room temperature. Gratifyingly, polymers **P5** and **P6** demonstrated *T*_g_’s well above room temperature, and surprisingly, polymers **P3** and **P8** also showed *T*_g_’s above room temperature. Among the polymers investigated, **P1** possesses a *T*_g_ below −80 °C,^22^ while **P6** exhibited a glass transition temperature of 104 °C. Thus, the polymers reported here encompass a wide range of glass transition temperatures spanning over 180 °C. These results underscore the flexibility and utility of the synthesis pathway developed in this study; the generality of the method enables the synthesis of monomers with a diverse array of structures, offering a versatile approach to producing polythioesters with highly tunable properties.

Additionally, biphenyl monomer **6** showed a low dispersity of 1.2. This surprising result is likely due to the lack of steric hindrance at the S_N_2 carbon due to the ring conformation of the monomer.^17^ By contrast, the conformation of the ring-opened system likely produces a more sterically hindered O–C carbon, reducing the favorability of backbiting or chain transfer reactions which increase dispersity. Macrocyclic monomer **7** also showed a relatively low dispersity of 1.4. Monomer **7** required modified conditions for polymerization (see the Supporting Information for a discussion), with the most important modification being a diluted 1 M polymerization concentration. Thus, the lower dispersity observed in this system is likely due to the lower concentration of chain ends relative to other systems.

While monomers **6** and **7** showed lower dispersities, no system investigated showed dispersities near 1.1 associated with highly controlled polymerization. Thus, possible sources of dispersity broadening were investigated. As thioacetates are generally understudied as both nucleophiles and leaving groups, it was unclear whether chain transfer reactions could occur readily in this system. To test the feasibility of chain transfer, a model thioester, *S*-dodecyl benzothioate **S5**, was treated with thioacetic acid and DBU in the previously discussed polymerization conditions (Figure S14). ^1^H NMR of the reaction after 3 h showed equilibration between the starting materials and the substituted products, *S*-dodecyl ethanethioate **S6** and thiobenzoic acid **S7**. This result indicates that chain transfer is possible in this system and is likely the primary side reaction which broadens dispersity during polymerization, as small molecule thiolactone products were not observed in high proportions by crude ^1^H NMR in many of the substrates indicating that backbiting reactions are minimal in most substrates. However, the greater driving force of the S_N_2 ROP reaction due to the C=S to C=O isomerization makes it unsurprising that propagation is preferred to chain transfer.

To further expand the substrate scope, polymerization of a new heterocyclic system was explored. Endo and co-workers previously reported cationic polymerization of thionocarbamates to the corresponding polythiocarbamates.^39,40^ Furthermore, isomerization of thionocarbamate monomer **S4** (Figure S11) to the corresponding thiocarbamate has been previously reported when **S4** was treated with iodide at 150 °C in xylenes.^41^ However, polymerization in analogous conditions (as well as the optimal conditions discussed earlier) was unsuccessful. It was hypothesized that one of three decomposition pathways could be occurring (Figure S13): (1) upon ring-opening, the thiocarbamate anion could irreversibly dissociate to gaseous carbonyl sulfide and an amine; (2) upon ring-opening, gaseous H_2_S could be expelled concomitant with isocyanate formation; or (3) the thionocarbamate could be deprotonated at the nitrogen to produce a resonance stabilized, non-nucleophilic anion. With these hypotheses in mind, alkylation of the nitrogen to produce monomer **8** was performed to limit decomposition pathways 2 and 3. Monomer **8** was shown to be polymerizable, demonstrating the generality of this polymerization pathway. In principle, other heteroatom systems which can release energy by undergoing X = S to X = O isomerization (such as cyclic thionophosphates) could also be polymerized via this reaction in the future.^42^

Finally, mild depolymerization of the product polythioesters was investigated (Figure 2). Previously, polymer **P2** was shown to be amenable to depolymerization.^25^ Polythioesters **P1**, **P4**, **P5**, and **P6** were successfully converted to the corresponding small molecule thiolactones with very high conversion upon treatment with catalytic quantities of TBD and dodecanethiol relative to the chain end (Figures S15–S18). It is hypothesized that dodecanethiol and TBD initiate a chain scission reaction via trans-thioesterification between dodecanethiol and the polymer backbone. The resulting thiolate chain end then continuously backbites to expel the corresponding small molecule thiolactones until depolymerization is complete. Isolated yields of between 50 and 88% for the corresponding thiolactones were obtained.

**Figure 2:**
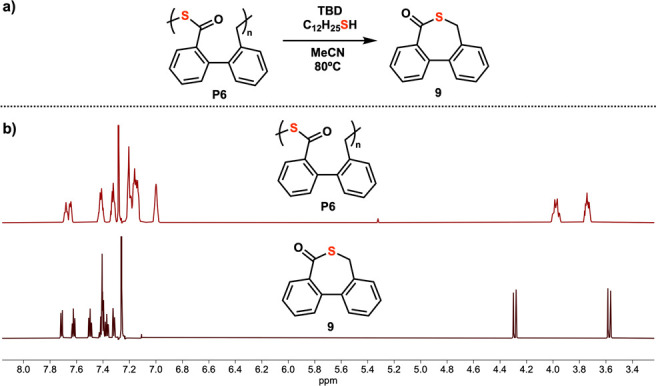
Depolymerization of polymers. (a) Example of depolymerization of polymer P6. Conditions: 1 equiv of **P6**, 20 equiv of TBD/dodecanethiol per chain end, 0.5 M with respect to moles of repeat units, MeCN, 80 °C, 2 h. (b) Comparison of ^1^H NMR of polymer **P6** compared to the thiolactone product **9** produced after depolymerization.

Depolymerization of the polythiocarbamate **P8** seemed to produce some of the corresponding small molecule by ^1^H NMR. The six-membered small molecule thiolactone **S8** which arises from depolymerization of **P1** has been demonstrated by our group to possess thermodynamics amenable to ROP—hinting at the possibility of employing the synthetic scheme developed here to produce polymers capable of chemical recycling to monomer

Overall, these results demonstrate that polymers with moderately sized repeat units (5–7 atoms) are well-suited for depolymerization, as they produce well-defined small molecule thiolactone products under mild depolymerization conditions. Many of these systems are kinetically trapped in the polymer state and thus are “spring-loaded” to depolymerize when the appropriate chemical trigger is added to the system. Additionally, the established processes of hydrolysis and aminolysis are applicable to polythioesters with any number of atoms in the repeat unit.^2,17^ Thus, there are multiple routes available for the conversion of polymers produced via this method to value-added chemicals at their end of life.

In conclusion, the exploration of reaction conditions and substrate scope reported here gives several useful insights into the production of polythioesters from lactone feedstocks. First, organic intuition based on the S_N_2 mechanism of polymerization illuminates the optimal reaction conditions: high concentration of monomer, polar aprotic environments, and an acid–base equilibrium strongly shifted toward the thioacetate anion. These insights lead to mild, inexpensive, and general conditions to accomplish this transformation. The utility of the method was demonstrated via the synthesis of polymers with glass transition temperatures spanning over 180 °C as well as the extension of this reaction to the polymerization of a thionocarbamate. Finally, the potential of these materials for use in applications requiring upcycling or recycling was demonstrated by mild depolymerization to well-defined small molecule products.

## Supporting Information Available

Experimental details and characterization data (PDF)
