# Supplementary material for: General and Mild Method for the Synthesis of Polythioesters from Lactone Feedstocks
Source: ACS Macro Lett. 2024 Oct 8;13(11):1411–7. doi: 10.1021/acsmacrolett.4c00556 (PMC11580380; doi:10.1021/acsmacrolett.4c00556)
Supplement: Supplementary file 1 — mz4c00556_si_001.pdf [file mz4c00556_si_001.pdf]

Supporting Information

General and Mild Method for the Synthesis of Polythioesters from Lactone Feedstocks

McKinley K. Paul, Matthew C. Raeside, and Will R. Gutekunst*

School of Chemistry and Biochemistry, Georgia Institute of Technology, Atlanta, Georgia 30332, United States

*Email: will.gute@chemistry.gatech.edu

TABLE OF CONTENTS

GENERAL PROCEDURES 2
SYNTHESES OF MONOMERS 3
INITIATOR SYNTHESIS AND NMR 4
INITIAL TESTS OF POLYMERIZABILITY 6
SOLVENT AND CONCENTRATION SCREENING 9
BACKBITING REACTION TO GENERATE THIOLACTONE SIDE PRODUCTS 11
INITIATOR SCREENING 12
SUBSTRATE SCREENING 13
UNSUCCESSFUL MONOMERS 15
CHAIN TRANSFER REACTION MODEL SYSTEM 16
DEPOLYMERIZATION 17
NMR CHARACTERIZATION OF POLYMERS 21
SEC CHARACTERIZATION 29
TGA 33
DSC 35
COMPUTATIONAL STUDIES 39
KINETIC STUDIES 41
REFERENCES 41

GENERAL PROCEDURES

All reactions were performed on a Schlenk line under nitrogen atmosphere with dry solvents using anhydrous conditions unless otherwise stated. All reaction vessels and stir bars were dried in an oven at 130ºC for at least 1h. Dry, degassed dichloromethane (DCM), tetrahydrofuran (THF), toluene, and dimethyl formamide (DMF) were obtained from a JC Meyer solvent purification system. Anhydrous, degassed dimethylacetamide was purchased from Thermofisher (Catalogue Number 044913.AE). Thioacetic acid was purchased from several vendors with that from Thermofisher found to perform optimally (Catalogue Number A0444782). Unless otherwise stated, all other reagents were purchased at the highest commercial quality and used without further purification. Yields refer to chromatographically and spectroscopically (^{1}H-NMR) homogeneous materials. Reactions were monitored by thin layer chromatography (TLC) carried out on 0.25 mm E. Merck silica gel plates (60F-254) using UV light, $KMnO_4$ or iodine vapor as visualizing agents. Silicycle silica gel (60 Å, particle size 0.043-0.063 mm) was used for flash column chromatography. NMR spectra were recorded on Bruker Avance 400, 500 or 700 MHz instruments and calibrated using residual undeuterated solvent as an internal reference ($CHCl_3$ at 7.26 ppm ^{1}H NMR, 77.16 ppm ^{13}C NMR), and then analyzed by MestReNova (version: 14.1.1). The following abbreviations (or combinations thereof) were used to explain the multiplicities: s = singlet, d = doublet, t = triplet, q = quartet, m = multiplet, br = broad. Polymerizations were typically performed in 2 mL vials (VWR, 46610-722). Polymer samples were analyzed using a Tosoh EcoSEC HLC 8320GPC system with TSKgel SuperHZ-L columns eluting $CHCl_3$ containing 0.25% NEt_3 at a flow rate of 0.45 mL/min at 40 ºC. All number-average molecular weights and dispersities were calculated from refractive index chromatograms using PStQuick Mp-M polystyrene standards unless otherwise stated. Thermogravimetric analyses (TGA) were performed under nitrogen atmosphere on TGA/DSC 3$^+$ STAR^e system (Mettler Toledo) at a heating rate of 10 °C/min. Differential scanning calorimetry (DSC) analyses were measured on a TA Instruments DSC250 under nitrogen atmosphere, and the reported data were obtained using a heating rate of 10 °C/min, unless otherwise stated.

SYNTHESES OF MONOMERS

Table S1. List of monomers used in this work and their references for synthesis.

Monomer Number	Structure	Name	Reference for Synthesis
**1**	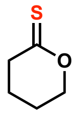	Thionovalerolactone	1
**2**	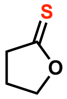	Thionobutyrolactone	2
**3**	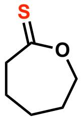	Thionocaprolactone	1
**5**	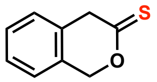	Thionoisochromanone	3
**6**	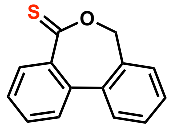	Dibenzo[*c*,*e*]oxepine-5(7*H*)-thione	4
**7**	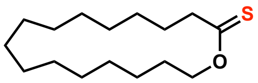	Thionohexadecanolide	1
**S1**	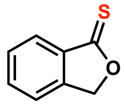	Thionopthalide	5
**S2**	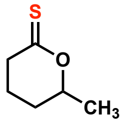	Delta-methyl thionovalerolactone	6

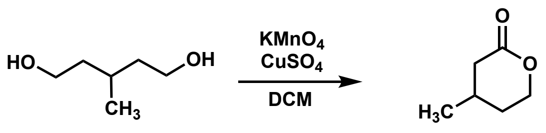

S3

4-methyltetrahydro-2*H*-pyran-2-one (S3): A suspension of $KMnO_4$ (25.9g, 163.8mmol, 6.3 eq) and $CuSO_4 \cdot 5\ H_2O$ (25.9g, 104mmol, 4 eq.) in dichloromethane (DCM) (127mL) was cooled to 0ºC. 3-methylpentane-1,5-diol (3.072g, 26mmol, 3.15mL, 1 eq.) was then added dropwise to this solution. The reaction was allowed to come to room temperature and stir for 24h. The reaction was then filtered through celite and triturated with DCM. The crude reaction (150ml) was then gently washed twice with aqueous Na_2SO_3 (50mL) . The organic phase was collected and solvent was removed in vacuo followed by drying overnight with high vacuum to yield the desired lactone **S3** (1g, 63% yield). Characterization of the product matched that of previous reports. [7]

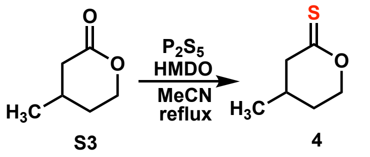

4-methyltetrahydro-2*H*-pyran-2-thione (Beta-Methyl Thionovalerolactone) (4): To a suspension of P_4S_{10} (1.285 g, 2.89 mmol, 0.25eq) in acetonitrile (MeCN) (7ml), was added hexamethyldisiloxane (HMDO) (3.38g, 20.8mmol, 4.42mL, 1.8eq). To this mixture, beta methyl lactone (**S3**) (1.32g,11.565mmol, 1.253mL, 1 eq.) was added. This reaction was heated to 80ºC for 6h. The reaction was then cooled to 0ºC and aqueous K_2CO_3 (1.26mL of 5.3M K_2CO_3 per mmol P_4S_{10}, or 2.7g K_2CO_3 in 3.64 mL deionized water) was added dropwise and allowed to react for 30 min. The reaction was diluted to a volume of 25mL with deionized water and extracted with EtOAc (3x50mL). Solvent was removed in vacuo and the crude product was purified via flash chromatography (50% Hexanes 50% EtOAc followed by a second column with 50% Et_2O 50% Hexanes) to yield **4** (685mg, 45% yield). Characterization data matched that from a previous literature report.[8]

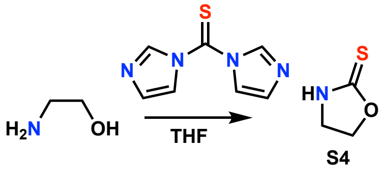

Oxazolidine-2-Thione (S4): A solution of ethanolamine (916mg, 15mmol, 0.90mL, 1 eq.) in tetrahydrofuran (THF) (10mL) was cooled to 0ºC. Thiocarbonyldiimidazole (TCDI) (2.67g, 15mmol, 1 eq.) was then added in portions. The reaction was allowed to come to room temperature and stirred for 3h. The solvent was removed in vacuo and purified via flash chromatography (80% EtOAc, 20% hexanes) to a yield **S4** (460mg, 30% yield). Characterization of the product matched that from a precious literature report.[9]

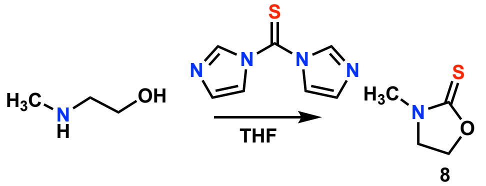

3-methyloxazolidine-2-thione (8): To a solution of 2-methylamino-1-ethanol (10g, 133mmol, 10.7mL, 1 eq.) in THF (98ml) at 0ºC was added thiocarbonyldiimidazole (TCDI) (23.726g, 133mmol, 1 eq.) in portions. The reaction was allowed to come to room temperature and react for 18 h. Solvent was removed in vacuo and the crude product purified via flash chromatography (100% EtOAc) to a yield **8** (6.7g, 67% yield). Characterization of the product matched that from a previous report.[10]

INITIATOR SYNTHESIS AND NMR

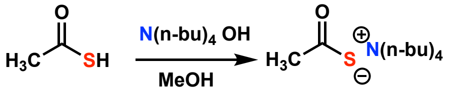

Tetrabutyl ammonium thioacetate: A methanolic solution of tetrabutylammonium hydroxide (5mL of 1M solution, 5mmol, 1297.49mg, 1 eq.) was degassed via sparging with nitrogen for 1 hour before cooling to 0ºC. Thioacetic acid (0.37mL, 399.6mg, 5.25mmol, 1.05eq) was then added dropwise while continuing to sparge the reaction with nitrogen. Reaction was allowed to come to room temperature and stir for 30 min before removing solvent in vacuo. The product was dried on high vacuum overnight to yield the product . The product was stored under nitrogen atmosphere refrigerator at 13ºC. ^1H NMR (400 MHz, $CDCl_3$) δ 3.43 – 3.34 (m, 8H), 2.52 (s, 2H), 1.75 – 1.62 (m, 10H), 1.46 (h, J = 7.4 Hz, 8H), 1.01 (t, J = 7.3 Hz, 11H). ^{13}C NMR (176MHz, $CDCl_3$) δ 216.27, 58.98, 39.54, 24.15, 19.82, 13.75.

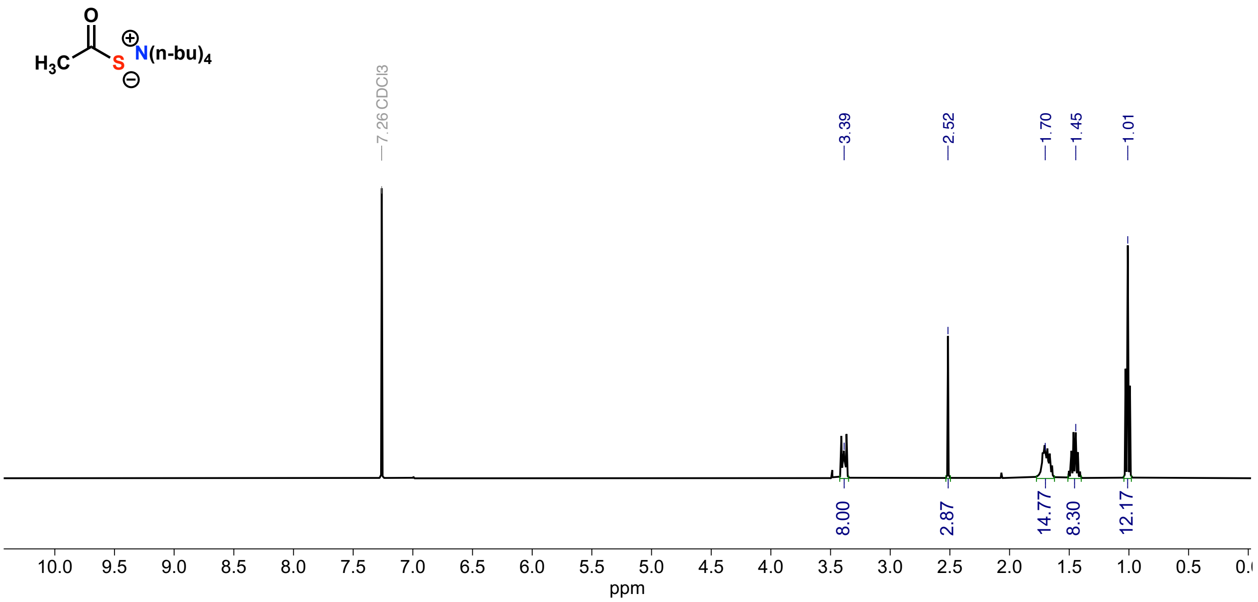

Figure S1. ^{1}H NMR ($CDCl_3$) of the tetrabutyl ammonium thioacetate salt. ^{1}H NMR (400 MHz, $CDCl_3$) δ 3.43 – 3.34 (m, 8H), 2.52 (s, 2H), 1.75 – 1.62 (m, 10H), 1.46 (h, J = 7.4 Hz, 8H), 1.01 (t, J = 7.3 Hz, 11H).

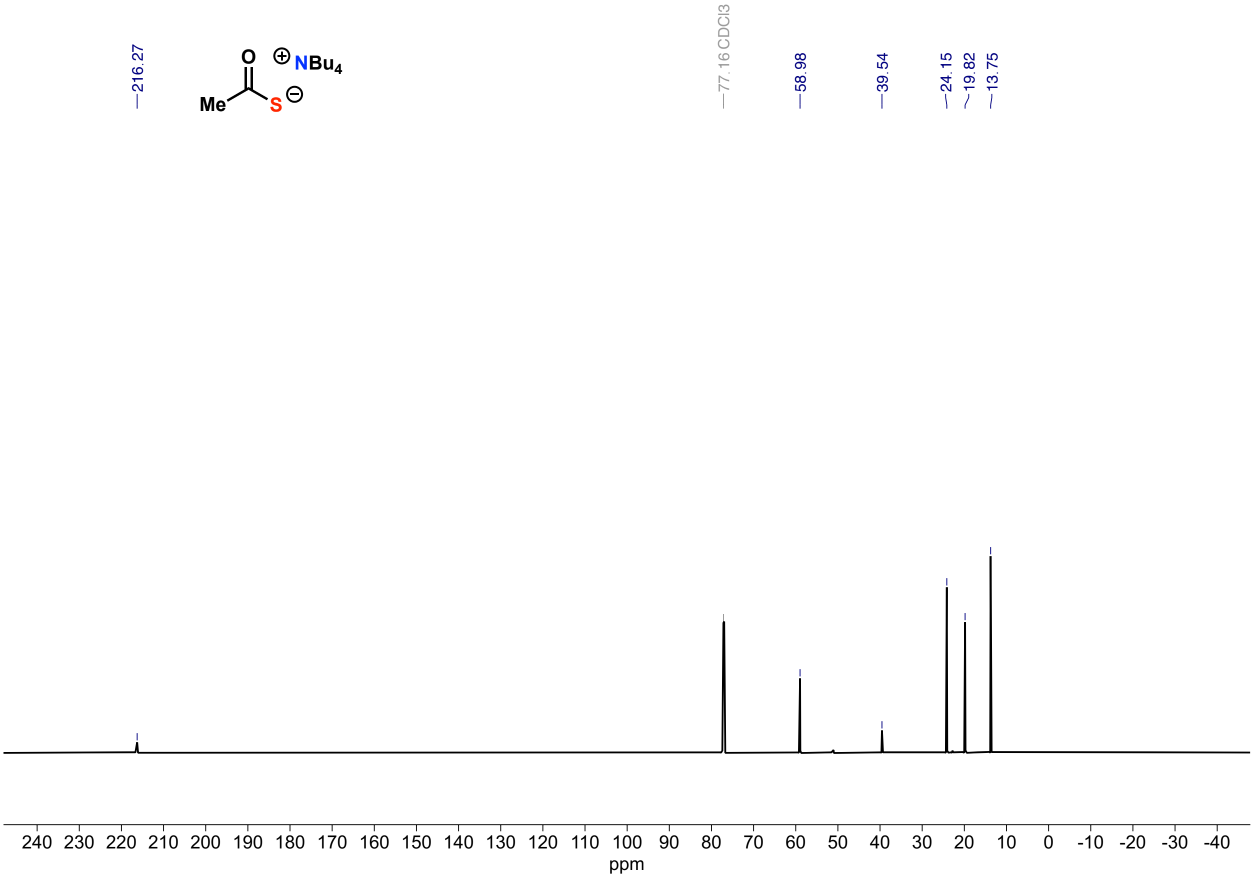

Figure S2. ^{13}C NMR of tetrabutyl ammonium thioacetate salt. ^{13}C NMR (176MHz, $CDCl_3$) δ 216.27, 58.98, 39.54, 24.15, 19.82, 13.75.

INITIAL TESTS OF POLYMERIZABILITY

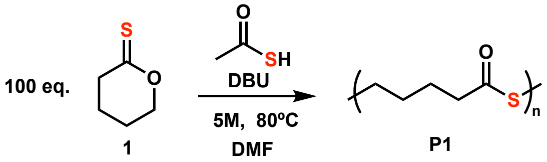

INITIAL TEST OF THIOACETATE INITIATORS: A 0.5M stock solution of thioacetic acid (0.5mmol, 38.06mg, 0.035mL) and DBU (0.5mmol,76.12mg, 0.0748mL) in DMF (0.89mL) was first prepared. Then, a solution of thionovalerolactone **1** (116.18mg, 0.1mL, 1mmol, 1 eq.) in DMF (76µL), was prepared and allowed to equilibrate to 80ºC for five minutes. The stock solution (20µL total volume containing 0.01 eq. of thioacetic acid and 0.01 eq. of DBU) was added to the monomer solution and allowed to react at 80ºC for 1h before being quenched with excess trifluoroacetic acid (TFA). The solution was then homogenized with $CDCl_3$ to obtain crude conversion data via NMR. The crude polymer was precipitated three times from DCM into methanol to remove low molecular weight products. ^{1}H NMR of the precipitated polymer: (700 MHz, $CDCl_3$) δ 2.87 (t, J = 7.2 Hz, 2H), 2.56 (t, J = 7.4 Hz, 2H), 1.77 – 1.68 (m, 2H), 1.64 – 1.57 (m, 2H). ^{13}C NMR of the precipitated polymer: (176 MHz, $CDCl_3$) δ 198.86, 43.33, 28.82, 28.27, 24.56.

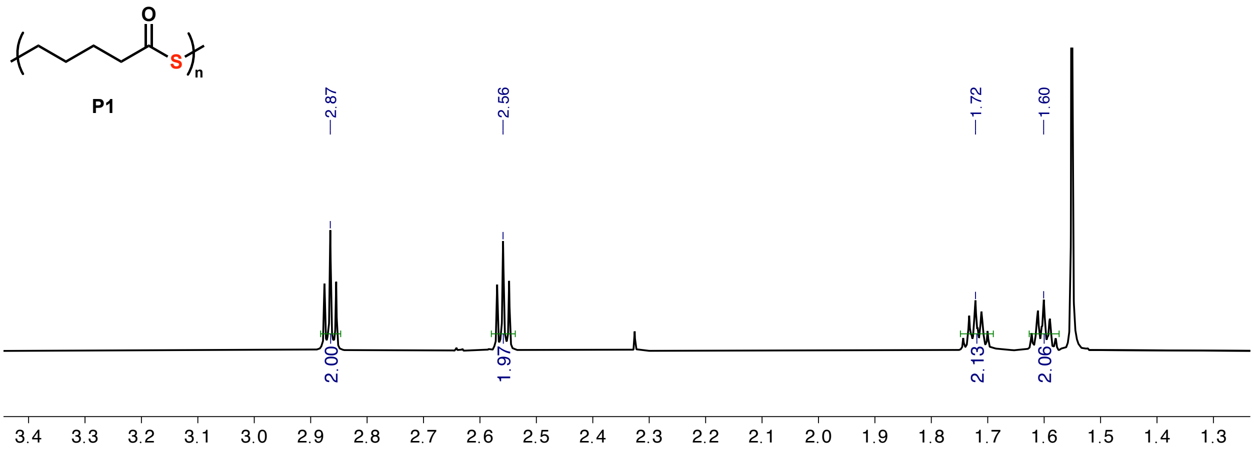

Figure S3. ^{1}H NMR ($CDCl_3$) of the precipitated polythioester **P1** prepared in the initial test of thioacetate initiators as described above. Conditions: 100 eq. thionovalerolactone, 1 eq. DBU, 1eq. thioacetic acid, DMF, 5M, 1h, 80ºC. ^{1}H NMR (700 MHz, $CDCl_3$) δ 2.87 (t, J = 7.2 Hz, 2H), 2.56 (t, J = 7.4 Hz, 2H), 1.77 – 1.68 (m, 2H), 1.64 – 1.57 (m, 2H).

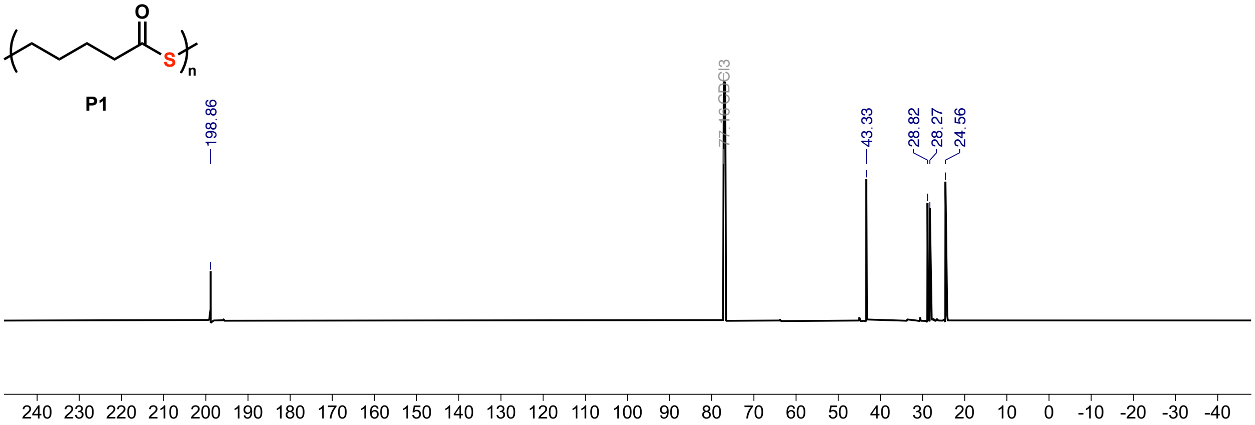

Figure S4. ^{13}C NMR ($CDCl_3$) of the precipitated polythioiester **P1** prepared in the initial test of thioacetate initiators as described above. Note the lack of the distinctive thionocarbonyl peak as seen in **Figure S4** at ~220ppm indicating the polymer is purely polythioester. Conditions: 100 eq. thionovalerolactone, 1 eq. DBU, 1eq. thioacetic acid, DMF, 5M, 1h, 80ºC. ^{13}C NMR (176 MHz, $CDCl_3$) δ 198.86, 43.33, 28.82, 28.27, 24.56.

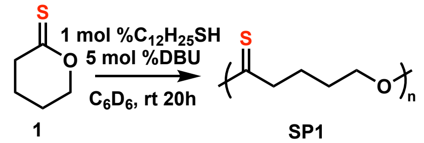

INDEPENDENT PREPARATION of POLYTHIONOESTER: Procedure adapted from Datta and Kiesewetter.[11] A stock solution of 0.125M DBU (0.125mmol, 19.03mg, 0.0187mL) and 0.025M dodecanethiol (0.025mmol, 5.06mg, 0.006mL) in dried and degassed C_6D_6 was prepared. Then, 0.272mL of this stock solution (containing 0.01 eq. of dodecanethiol and 0.05 eq. of DBU) was added to thionovalerolactone **1** (81.326mg, 0.7mmol, 0.070mL, 1 eq.) and allowed to react for 20h at room temperature before quenching with TFA. The polymer product **SP1** was purified via precipitation from DCM into MeOH 3x. ^{1}H NMR of the precipitated polymer: (500 MHz, $CDCl_3$) δ 4.45 (t, J = 6.1 Hz, 2H), 2.76 (t, J = 7.1 Hz, 2H), 1.93 – 1.77 (m, 4H). ^{13}C NMR of the precipitated polymer: (176 MHz, $CDCl_3$) δ 223.41, 72.08, 46.24, 27.41, 25.07.

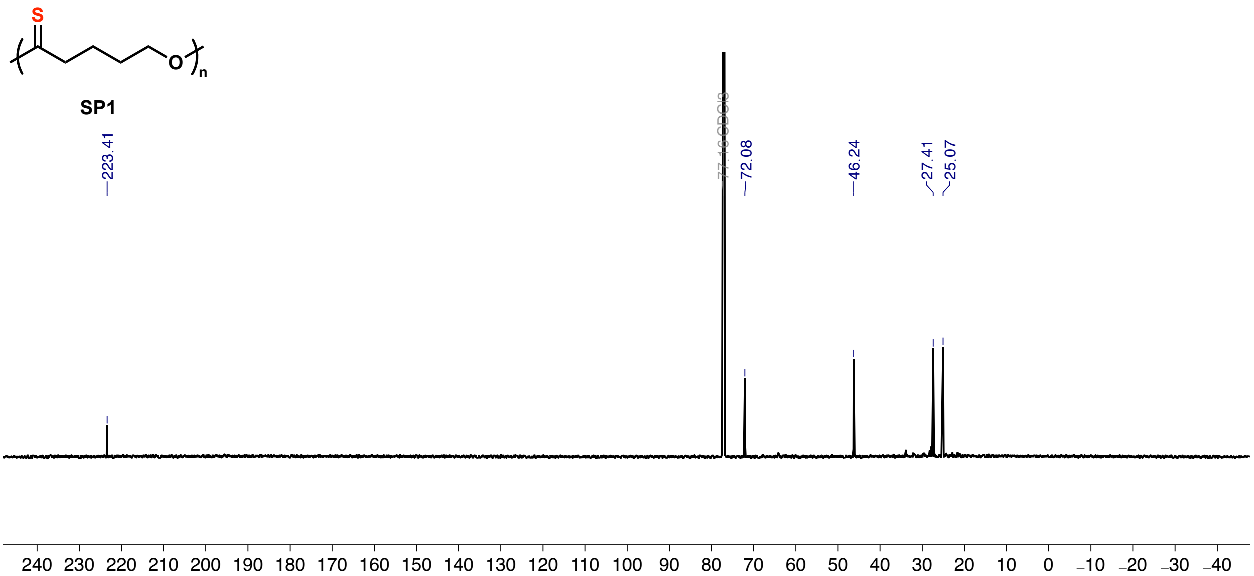

Figure S5. ^{13}C NMR ($CDCl_3$) of the precipitated polythionoester polymer **SP1** synthesized as described above. Note the distinctive thionocarbonyl peak at 223ppm. Conditions: 100 eq. Thionovalerolactone, 1 eq. dodecanethiol, 5 eq. DBU, 2M in C_6D_6, r.t. 20h. ^{13}C NMR (176 MHz, $CDCl_3$) δ 223.41, 72.08, 46.24, 27.41, 25.07.

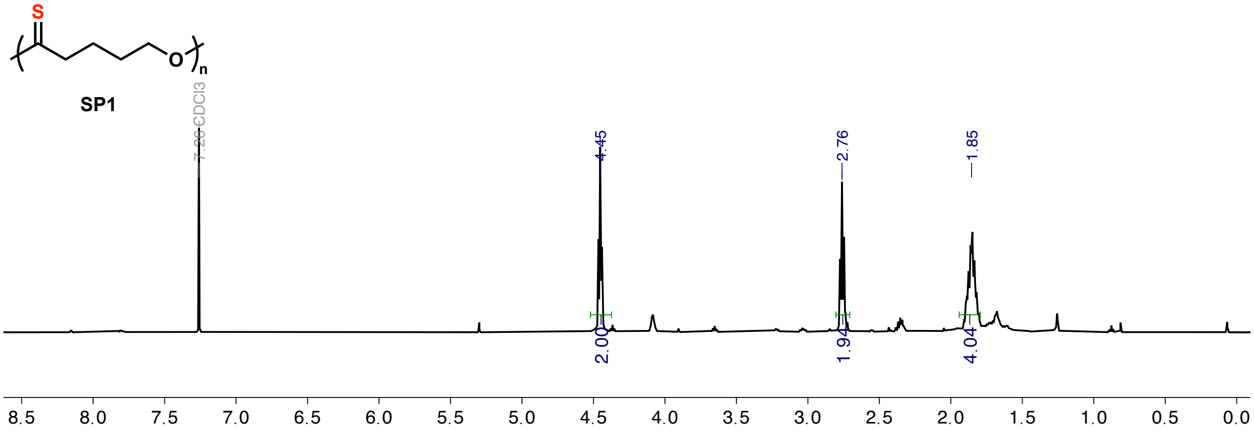

Figure S6. ^{1}H NMR ($CDCl_3$) of the precipitated polythionovalerolactone **SP1** synthesized as described above. Conditions: 100 eq. Thionovalerolactone, 1 eq. dodecanethiol, 5 eq. DBU, 2M in C_6D_6, r.t. 20h. ^{1}H NMR (500 MHz, $CDCl_3$) δ 4.45 (t, J = 6.1 Hz, 2H), 2.76 (t, J = 7.1 Hz, 2H), 1.93 – 1.77 (m, 4H).

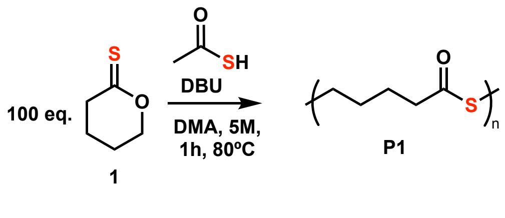

POLYMERIZATION WITH DIMETHYLACETAMIDE (DMA) IN PLACE OF DMF: Polymerization was carried out as described in the initial test of thioacetate initiators, except with DMA as solvent.

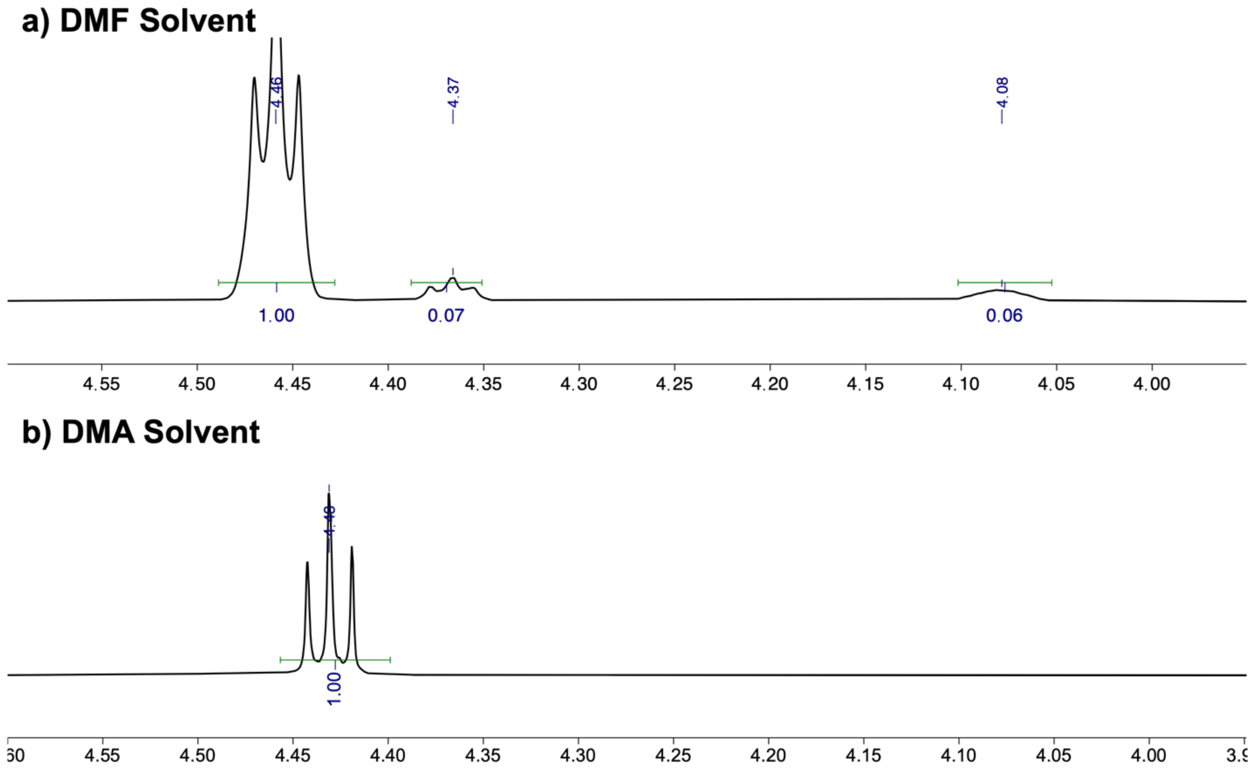

Figure S7. ^{1}H NMR ($CDCl_3$) Comparison of polymerization of thionovalerolactone **1** to polythioester **P1** with (a) DMF as solvent and (b) DMA as solvent. Note the lack of side products in DMA present around 4.37 and 4.08ppm. Conditions: 100 eq. thionovalerolactone, 1 eq. DBU, 1eq. thioacetic acid, 5M, 1h, 80ºC.

SOLVENT AND CONCENTRATION SCREENING

Following the initial success with DMA as solvent, other solvents were screened. (**Table S2** and **Figure S8**). More polar solvents were expected to accelerate the S_N2; thus, MeCN was also screened as solvent. However, DMA showed significantly superior performance. Toluene, the solvent employed by Hong and coworkers in both their anionic and cationic studies,[2,12] was found to perform poorly in this system. As the thioacetate anion has previously been hypothesized to engage in hydrogen bonding,[2,13] solvents capable of hydrogen bonding were screened. On the grounds that engaging the thioacetate chain could potentially reduce dispersity broadening backbiting and chain transfer reactions, tert-butanol (^{t}BuOH) was investigated as a relatively non-nucleophilic hydrogen bond donor. Additionally, dioxane was also screened due to its potential to engage the DBU-H conjugate acid in hydrogen bonding. However neither ^{t}BuOH nor dioxane seemed to improve the conversion or control of the polymerization.

Table S2. Solvent screening. Conditions: 100 eq. thionovalerolactone, 1 eq. DBU, 1eq. thioacetic acid. 3M, 3h, 80ºC. Quenched with excess TFA

	DMA	MeCN	Toluene	Dioxane	tBuOH
**Attribute**	Polar	Polar	Nonpolar	H-Bond Acceptor	H-Bond Donor
**Conversion**	87%	35%	32%	28%	30%
**Dispersity**	1.64	2.16	1.75	1.45	1.88
**M_w (g/mol)**	8,955	7,560	5,046	4,341	6,846
**DP**	47	30	25	26	31

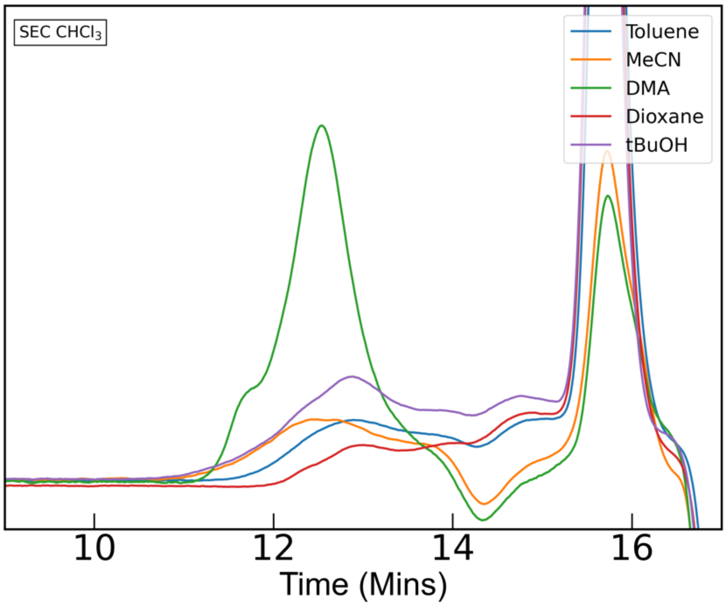

Figure S8. $CHCl_3$ SEC of solvent screening experiments of **Table S2**.

The effect of concentration was also investigated (**Table S3** and **Figure S9**). While increasing concentration tended to improve conversion, no clear trend in molecular weight or dispersity was shown. While the polymerization was shown to be viable in the with minimal solvent at high temperature in the bulk monomer, this polymerization showed significantly broader dispersity and thus was not pursued further. Among the concentrations screened, 5M concentration showed the best performance, thus it was chosen when evaluating novel substrates.

Table S3. Concentration screening. Conditions: 100eq. thionovalerolactone, 1 eq. thioacetic acid, 1eq. DBU, DMA, 80ºC, 3h. Quenched with TFA. a) bulk 115ºC, 30min.

Concentration	Conversion	Dispersity	M_w (g/mol)	DP
3M	87%	1.6	8,955	47
4M	82%	2.12	11,369	46
5M	95%	1.78	9,478	46
8.3M[a]	98%	2.1	11,608	48

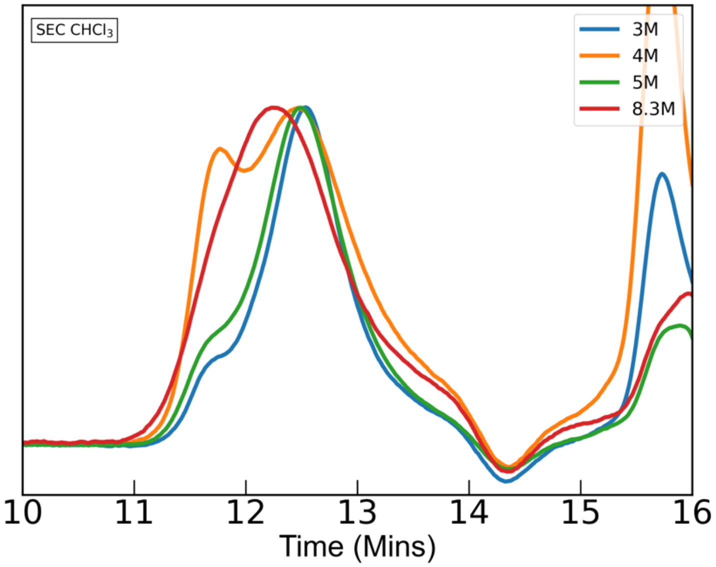

Figure S9. SEC $CHCl_3$ of concentration screening in **Table S3.**

BACKBITING REACTION TO GENERATE THIOLACTONE SIDE PRODUCTS

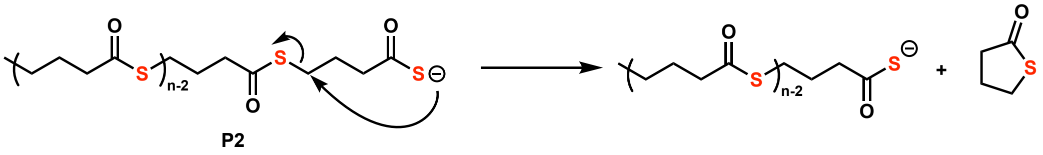

Figure S10. Example of backbiting side reaction to generate small molecule thiolactone products. Backbiting is shown for polymer **P2**.

INITIATOR SCREENING

GENERAL INITIATOR SYSTEM SCREENING PROCEDURE

Under N_2, the thionovalerolactone monomer **1** (100eq., 0.1mL, 116mg, 1mmol) was dissolved in DMA (0.18mL) in an oven dried 2mL vial and heated to 80ºC and allowed to equilibrate for 5min. A stock solution of the initiator system in DMA was prepared. 20µL of this stock solution was added to the reaction vial containing monomer and allowed to react for 3h at 80ºC before being removed from the oil bath for several minutes to cool. The reaction was subsequently quenched with 2-3 drops of TFA. The polymerization was homogenized with $CDCl_3$ and aliquot of this crude sample was analyzed via ^{1}H NMR to determine conversion. The polymer was then purified via precipitation from DCM into MeOH 3x. The precipitated polymer was then subjected to ^{1}H NMR and SEC.

Table S4. Results of initiator system screening using general procedure above.

Entry	Initiator System	Conversion	DP	Dispersity
**1**	1 eq. Thioacetic Acid + 1 eq. DBU	95%	46	1.78
**2**	1 eq. DBU	96%	30	2.21
**3**	1 eq. BnSH + 1 eq. DBU	95%	29	2.04
**4**	1eq. Diphenyl methanol + 1 eq. DBU	93%	32	2.07
**5**	1 eq. Thioacetic Acid + 1 eq. DBU + 2 eq. Hexafluoroisopropanol	72%	15	1.86
**6**	1 eq. Thioacetic Acid + 1 eq. DBU + 1 eq. Diphenyl Methanol	89%	27	1.79
**7**	1 eq. Thioacetic Acid + 1 eq. Hunig's base	61%	13	1.79
**8**	1 eq. Thioacetic Acid + 1 eq. 2,6-lutidine	trace	--	--
**9**	1 eq. Thioacetic Acid + 1 eq. quinuclidine	48%	11	1.81
**10**	1 eq. $[CH_3COS]^-$ $[N(n\text{-}Bu)_4]^+$	99%	98	1.62

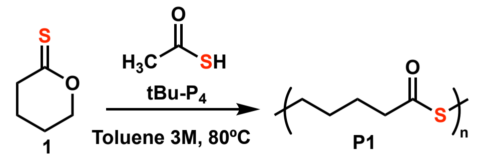

SCREENING OF PHOSPHAZENE SUPERBASE: The combination of thioacetic acid and phosphazene superbase were screened in conditions similar to those previously reported as follows:[2] Under N_2, anhydrous, degassed toluene (0.12mL) was added to a vial containing thionovalerolactone monomer **1** (0.6mmol, 69.7mg, 0.0606mL, 100 eq.). The solution was then heated to 80ºC and allowed to equilibrate for ten minutes. A stock solution of the phosphazene superbase (0.1mmol,63.37mg, 0.1mL of a 0.8M solution of $tBuP_4$ in hexanes) and thioacetic acid (0.1mmol,7.6mg, 7μL) in toluene (0.226mL) was prepared. Stock solution (20μL containing 1eq. thioacetic acid and 1eq. tBu-P_4) was added to the monomer solution. The reaction proceeded for 0.5h at 80ºC before it was quenched with TFA. Solution was homogenized with $CDCl_3$ and an aliquot of this solution was submitted to ^{1}H NMR to determine conversion. The polymer was then precipitated from DCM into methanol three times before being submitted to ^{1}H NMR and SEC. Reaction produced a conversion of 96%, DP of 26 and dispersity of 1.70.

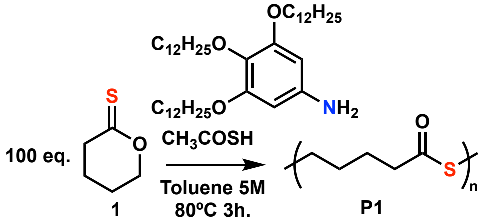

SCREENING OF 3,4,5-TRIS(DODECYLOXY) ANILINE: 3,4,5-tris(dodecyloxy) aniline (64.61mg, 0.1mmol) added to a vial and placed under nitrogen atmosphere. Toluene (0.193mL) and thioacetic acid (0.1mmol, 7.6mg, 7μL) were added to the 3,4,5-tris(dodecyloxy) aniline to produce a stock solution. In a separate vial, thionovalerolactone **1** (116.18mg, 1mmol. 0.1mL, 100eq.) was added to a vial containing 0.08mL toluene. Stock solution (20μL containing 1 eq. 3,4,5-tris(dodecyloxy) aniline and 1 eq. thioacetic acid) was added to the monomer. The reaction was heated to 80ºC and allowed to react for 3 hours before quenching with several drops of TFA. Solution was homogenized with $CDCl_3$ and an aliquot of this solution was submitted to ^{1}H NMR to determine conversion. The polymer was then precipitated from DCM into methanol three times before being submitted to ^{1}H NMR and SEC. Reaction produced a conversion of 10%, DP of 60 and dispersity of 2.03.

SUBSTRATE SCREENING

GENERAL OPTIMIZED POLYMERIZATION PROCEDURE

As the thioacetate anion is known to disproportionate to acetate and sulfur in the presence of oxygen,[13] performing the polymerization under inert atmosphere with degassed, anhydrous, and purified reagents is essential for obtaining optimal performance. Accordingly, anhydrous DMA was distilled over CaH_2 and stored in a Schlenk flask under nitrogen and activated 4Å molecular sieves. To begin the polymerization, under N_2, the thionolactone monomer (200eq., 1mmol) was dissolved in DMA (to achieve a total concentration of 5M of monomer in the reaction including the volume of stock solution to be added) in an oven dried 2mL vial and heated to 80ºC and allowed to equilibrate for 5min. A stock solution of tetrabutyl ammonium thioacetate initiator (10mg, 0.031mmol) in DMA (0.126mL) was prepared. 20μL of this stock solution (containing 0.005mmol, 1 eq. initiator) was added to the reaction vial containing monomer and allowed to react at 80ºC for the specified reaction time (**Table S5**) before being removed from the oil bath for several minutes to cool. The reaction was subsequently quenched with 2-3 drops of TFA. The polymerization was homogenized with $CDCl_3$ and an aliquot of this crude sample was analyzed via ^{1}H NMR and Size Exclusion Chromatography (SEC). The polymer was then purified via precipitation from DCM into MeOH 3x. If insoluble in DCM, the polymer was dissolved in hot $CHCl_3$ and subsequently

precipitated into room temperature MeOH 3x for purification. The precipitated polymer was then subjected to ^{1}H NMR, ^{13}C NMR, SEC, TGA, and DSC. Spectral characterization of the previously synthesized polymers **P1**[14], **P2**[2], and **P3**[15] matched that of previous reports. Tabulated NMR data for newly synthesized polymers can be found in the **NMR CHARACTERIZATION OF POLYMERS** section in the figure caption for each spectra.

Table S5. Table of polymers synthesized via the optimized general procedure.

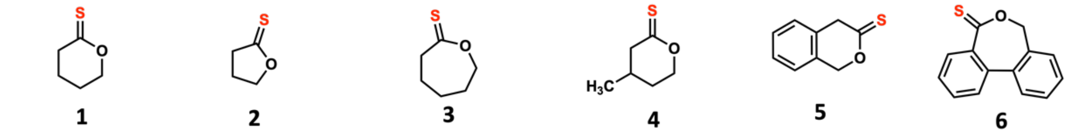

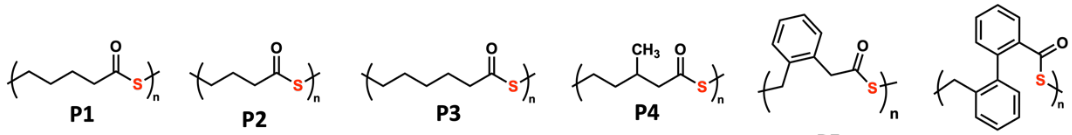

**Monomer**	**1**	**2**	**3**	**4**	**5**	**6**
**Polymer**	**P1**	**P2**	**P3**	**P4**	**P5**	**P6**
**M_W (g/mol)**	21,500	42,500	41,100	32,700	5,800	40,900
**Dispersity**	1.5	1.8	2.2	1.5	2.0	1.2
**Conversion**	98%	99%	98%	81%	96%	99%
**Reaction Time**	3h	1.5h	22h	3h	4.5h	0.75h

Note on the Polymerization of Thionohexadecanolide 7: It was found that macrocyclic monomer **7** did not polymerize when treated with tetrabutyl ammonium thioacetate initiator in DMA, but instead polymerized when treated with the DBU and thioacetic acid initiator system in *N,N*'-dimethylpropylen-urea (DMPU) solvent. Monomer **7** required higher temperatures to induce polymerization as well as lower concentrations of monomer. As the monomer is a nonpolar liquid with a very low density, a solution of this monomer at 3 M concentration is almost completely monomer by volume. Thus, concentrated solutions of this monomer provide environments that are not polar enough to stabilize the S_N2 reaction, and the monomer must be diluted with the more polar solvent to yield an environment that is polar enough for the S_N2. In practice, best results were found with a 1 M solution of monomer in DMPU.

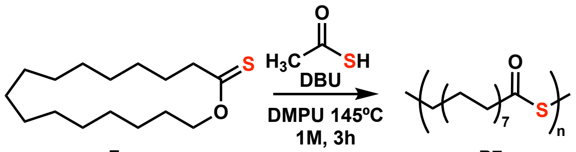

Polythiohexadecanolide P7: DMPU was dried over activated molecular sieves for 4 days before being degassed via sparging with nitrogen for 30min. Thionohexadecanolide (**7**) (0.9mmol, 0.25mL, 243.43mg, 1 eq) was mixed with 0.63mL of DMPU. A 0.225M stock solution of thioacetic acid (0.25mmol, 19.03mg, 0.0176mL) and DBU (0.25mL, 38.06mg, 0.0374mL) in DMPU(1.056mL) was prepared. The stock solution was degassed via sparging with nitrogen for 10 minutes. Stock solution (0.02mL of stock solution containing 0.0045mmol, 0.005eq. of DBU and thioacetic acid each) was then added to the monomer solution. The reaction was placed in an oil bath that was preheated to 145ºC and allowed to react for 3h.The solution then was removed from heat and allowed to cool for several minutes before a solution of TFA/$CDCl_3$ was added to the reaction to quench. Reaction solution was homogenized in $CDCl_3$ before being submitted to ^{1}H NMR which showed that the reaction proceeded to 78% conversion. The polymer was then precipitated twice from 55ºC chloroform into room

temperature methanol before being submitted to ^{1}H NMR, ^{13}C NMR, SEC, TGA, and DSC. Mw = 51,600, Đ = 1.4 for the precipitated polymer.

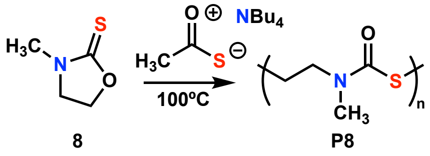

Poly-3-methyloxazolidine-2-thione P8: Tetrabutyl ammonium thioacetate (5.40mg,0.017mmol,0.005eq) was added to an oven dried vial containing monomer **8** (398mg, 3.4mmol, 1 eq.). The reaction vial was placed under nitrogen atmosphere and heated to 100°C (at 44°C monomer **8** melts) and allowed to react for 1h. The reaction was allowed to cool before being quenched with 1 drop of TFA in hexafluoroisopropanol (HFIP). Reaction proceeded to 69% conversion as determined by crude ^{1}H NMR. Polymer **P8** was then precipitated twice from hot HFIP into room temperature solution of 95% hexanes/5% EtOAc before being submitted to ^{1}H NMR, ^{13}C NMR, SEC, TGA, and DSC. Mw = 29,300, Đ = 1.4 for the precipitated polymer.

UNSUCCESSFUL MONOMERS

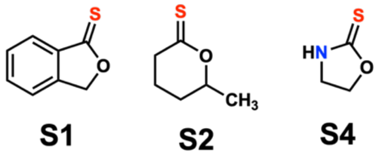

Figure S11. Monomers unable to be polymerized with tetrabutylammonium thioacetate initiators.

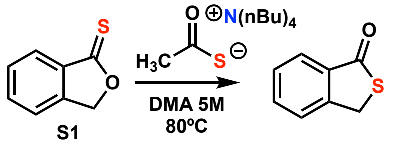

Figure S12. Small molecule thiolactone product observed when thionophthalide monomer **S1** was treated with tetrabutyl ammonium thioacetate. Characterization of the thiolactone product matched that of previous reports.[13]

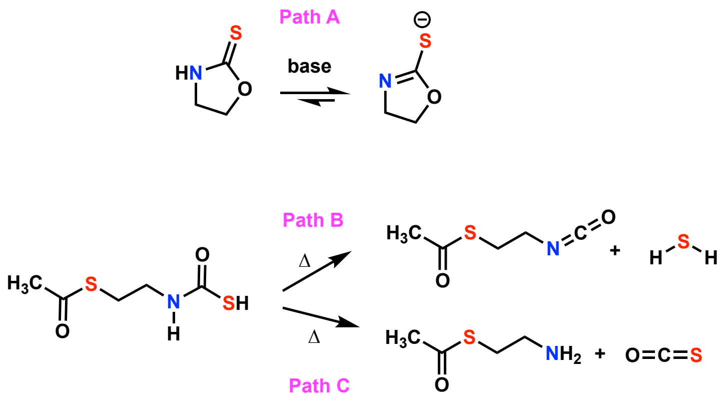

Figure S13. Possible decomposition pathways of monomer **S4** when treated with tetrabutyl ammonium thioacetate initiator. Path A represents deprotonation to generate a resonance stabilized and potentially non-nucleophillic anion. Path's B and C represent possible decomposition pathways after an initial ring opening

event. Alkylation of the nitrogen atom (as in monomer **8**) was expected to discourage decomposition pathways A and B.

CHAIN TRANSFER REACTION MODEL SYSTEM

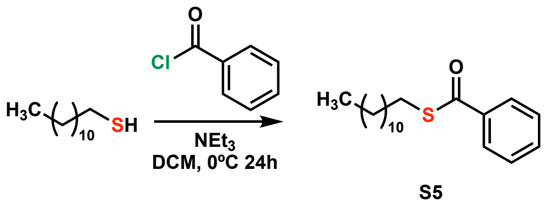

SYNTHESIS OF S-DODECYL BENZOTHIOATE S5. **S5** was synthesized according to a modified literature procedure.[16] To a solution of benzoyl chloride (1.2 eq., 3.6mmol, 0.42mL) in DCM (1.3mL) was added dropwise a solution of dodecanethiol (1 eq., 3mmol, 0.72mL) and triethylamine (1.2 eq., 3.6mmol, 0.5mL) in DCM (1.3mL) at 0ºC. The reaction mixture was allowed to come to room temperature and proceed for 12h purification of the crude mixture via column chromatography. Characterization of the product matched that of literature reports.

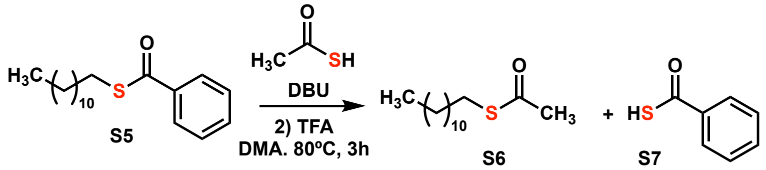

CHAIN TRANSFER MODEL REACTION: Thioacetic Acid (1 eq. 1mmol, 76.11mg, 0.0705mL) was added to a solution of S-dodecyl Benzothioate **S5** (1 eq. 1mmol, 306.51mg) in DMA (0.45mL). DBU (1 eq. 1mmol. 0.15mL) was then added to the reaction mixture. Reaction was heated to 80ºC and allowed to proceed for 3h before quenching with TFA (1.5eq.) The crude reaction mixture was then analyzed by ^{1}H NMR as shown in **Figure S14(c)**. New peaks observed at 7.91, 7.60 and 7.41ppm corresponding to thiobenzoic acid product **S7**, indicate that the thioacetate anion is able to perform an S_N2 reaction of the model substrate as shown in **Figure S14(b).** Integration indicates that 27% of **S5** was converted into **S6.** This result indicates that chain transfer reactions are feasible in the polymerization system reported here, and thus are likely a source of dispersity in any of the reported polymerizations.

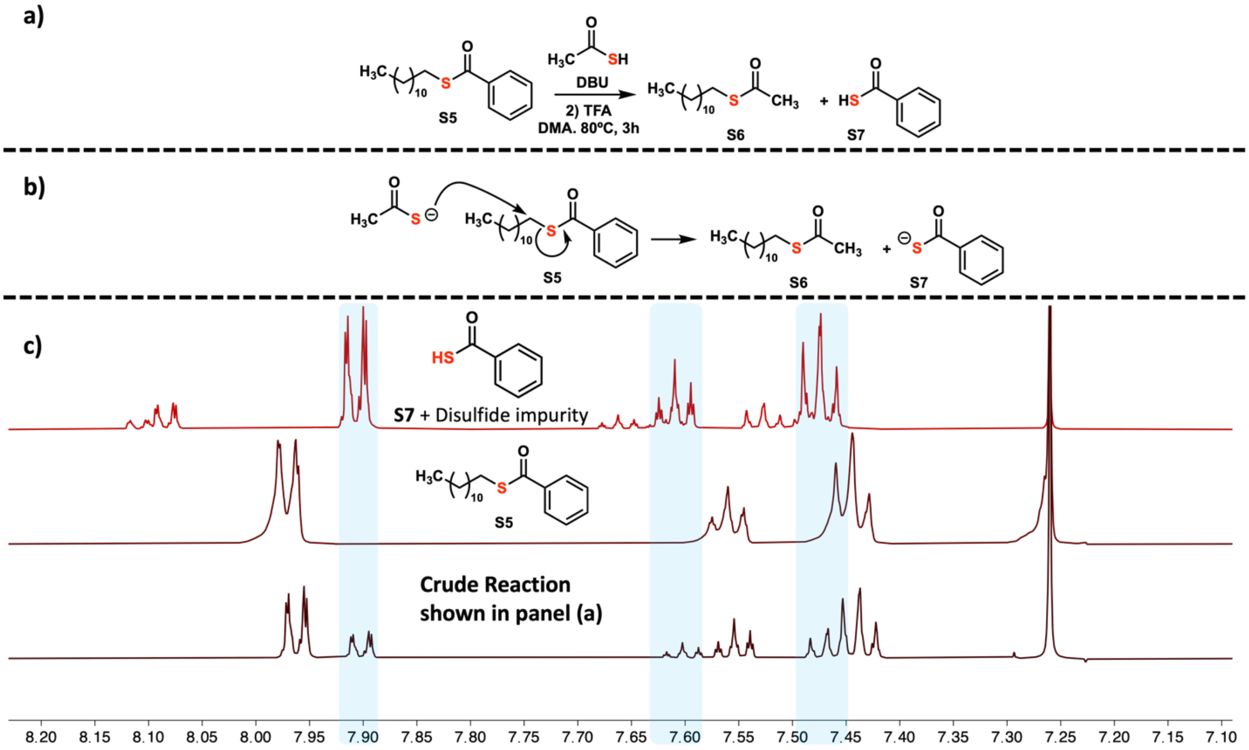

Figure S14. a) Reaction scheme of model reaction. (b) Proposed mechanism of substitution and chain transfer. (c) ^{1}H NMR (CDCl$_3$) comparison of starting material **S5** to the crude reaction and product **S7**. Blue highlighted peaks at 7.91,7.60, and 7.41ppm indicate formation of product **S7**.

DEPOLYMERIZATION

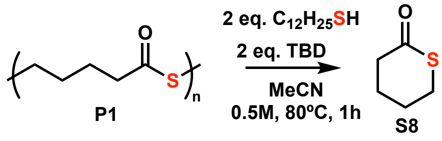

DEPOLMYERIZATION OF P1: P1 (M_n = 5,273g/mol, DP=90.36, D=1.99) (105.46mg, 0.02mmol, 1 eq.) was added as a solid to a vial containing triazabicyclodecene (TBD) (5.56mg, 0.04mmol, 2 eq. relative to the number of moles of polymer chains based on SEC measured polymer M_n). The polymer and TBD were placed under nitrogen atmosphere and dodecanethiol (10µL, 8mg, 0.04mmol, 2 eq.) was added to the reaction vial followed by MeCN (3.6mL, volume chosen to yield a 0.5M theoretical concentration of thiolactone product). The reaction was heated to 80ºC and ran for one hour. After removing heat and allowing the reaction to cool for several minutes, several drops of TFA were added to quench the reaction. Crude ^{1}H NMR indicated that the reaction proceeded to full conversion. The crude thiolactone product **S8** was purified via flash chromatography (50% Et_2O, 50% hexanes) (92mg, 88% yield). ^{1}H NMR (400 MHz, CDCl$_3$) δ 3.16 (ddd, J = 6.5, 5.0, 1.1 Hz, 1H), 2.66 – 2.56 (m, 1H), 2.07 – 1.92 (m, 2H). ^{1}H NMR of the product matched that of previous reports;[14] however, impurities in the 1.90ppm-0.70ppm region were unable to be removed via flash chromatography.

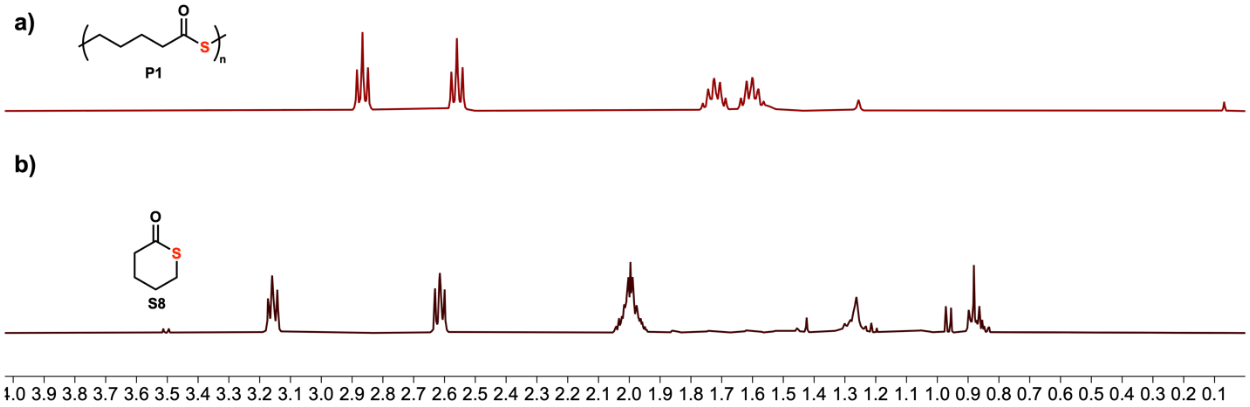

Figure S15. Comparison of **P1** before and after depolymerization. (a) ^{1}H NMR ($CDCl_3$) of **P1** (b) ^{1}H NMR ($CDCl_3$) of thiolactone product **S8**.

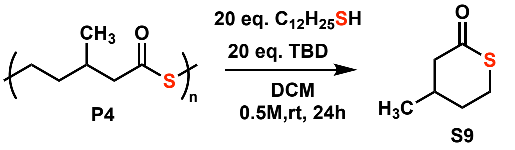

DEPOLYMERIZATION OF P4: P4 (M_n = 21,629, DP = 166, D = 1.51) (62.5 mg, 1 eq., 0.00289mmol) was added to a vial containing TBD (8mg, 0.0578mmol, 20 eq. relative to the number of moles of polymer chain ends based on SEC measured polymer M_n). The reaction vial was placed under nitrogen atmosphere and dichloromethane (DCM) was added (0.96mL, volume chosen to yield a theoretical 0.5M concentration with respect to the thiolactone product). Dodecanethiol (14µL, 11.7mg, 0.0578mmol, 20 eq.) was added and the reaction was stirred for 24h at room temperature before quenching with TFA (0.013mL). Crude ^{1}H NMR indicated that the reaction proceeded to 90% conversion. The crude product was dissolved into EtOAc and washed with once with 1N HCl and once with brine. The organic phase was collected and solvent removed en vacuo. The product was then further purified by preparatory TLC (30% EtOAc, 70% Hexanes) (46.85mg, 75% yield). ^{1}H NMR (400 MHz, $CDCl_3$) δ 3.21 (ddd, J = 12.4, 10.6, 4.4 Hz, 1H), 3.11 (dt, J = 12.4, 4.8 Hz, 1H), 2.67 (ddd, J = 16.9, 4.1, 1.3 Hz, 1H), 2.29 – 1.99 (m, 3H), 1.60 (dddd, J = 13.5, 10.7, 9.8, 4.6 Hz, 1H), 1.06 (d, J = 6.7 Hz, 3H). ^{1}H NMR matched that of previous reports.[14]

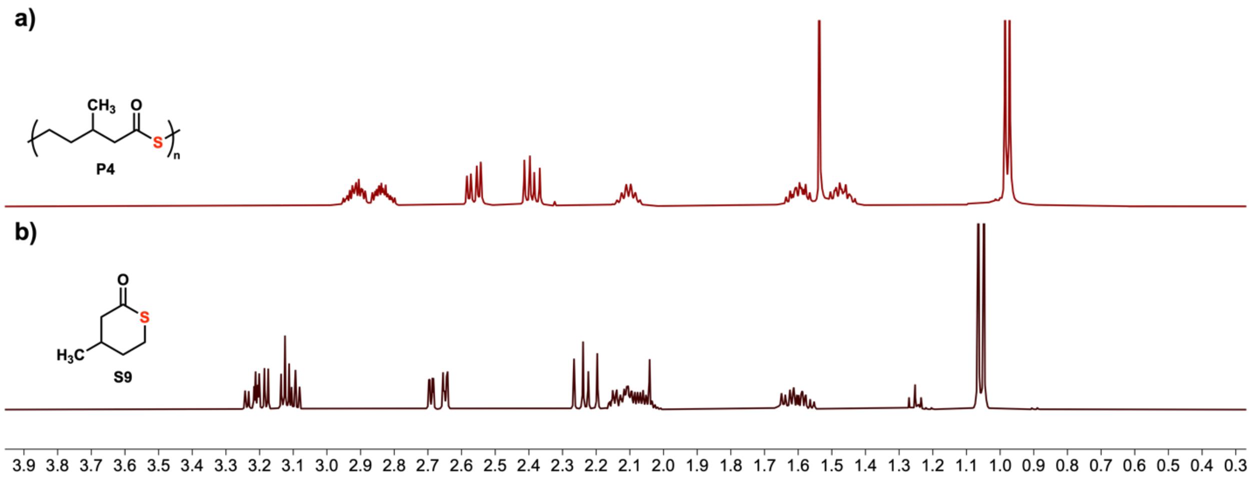

Figure S16. Comparison of **P4** before and after depolymerization. (a) ^1H NMR ($CDCl_3$) of **P4** (b) ^1H NMR ($CDCl_3$) of thiolactone product **S9**.

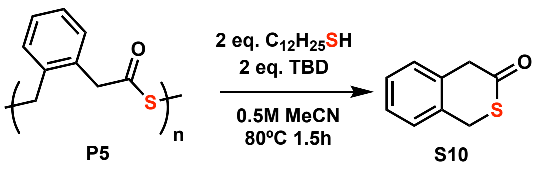

DEPOLYMERIZATION OF POLYMER P5: **P5** (M_n = 5,758, DP = 17.48, Đ = 2.006) (224.7mg, 0.039mmol, 1 eq.) was added to a vial containing TBD (11mg, 0.078mmol, 2 eq. relative to the number of moles of polymer chain ends based on SEC measured M_n). The vial was placed under nitrogen atmosphere and MeCN (2.74mL, volume chosen to produce a theoretical 0.5M concentration of thiolactone product) was added followed by dodecanethiol (0.0187mL, 15.8mg, 0.078mmol, 2eq.). The reaction was heated to 80°C for 1.5h. The reaction was then removed from heat for several minutes and allowed to cool before several drops of TFA were added to quench the reaction. Crude ^1H NMR indicated that the reaction proceeded to full conversion. The product was dissolved in 100mL EtOAc and washed twice with 50mL of 1N HCl and once with 50mL brine. The organic phase was collected and solvent removed in vacuo. The product was then purified by flash chromatography (25% EtOAc/75% Hexanes) (178.5mg, 80% yield). ^1H NMR (500 MHz, $CDCl_3$) δ 7.31 (dt, J = 19.3, 4.6 Hz, 3H), 7.22 (q, J = 4.9 Hz, 1H), 4.23 (s, 2H), 3.80 (s, 2H). Characterization of the product matched that previous reports.[18]

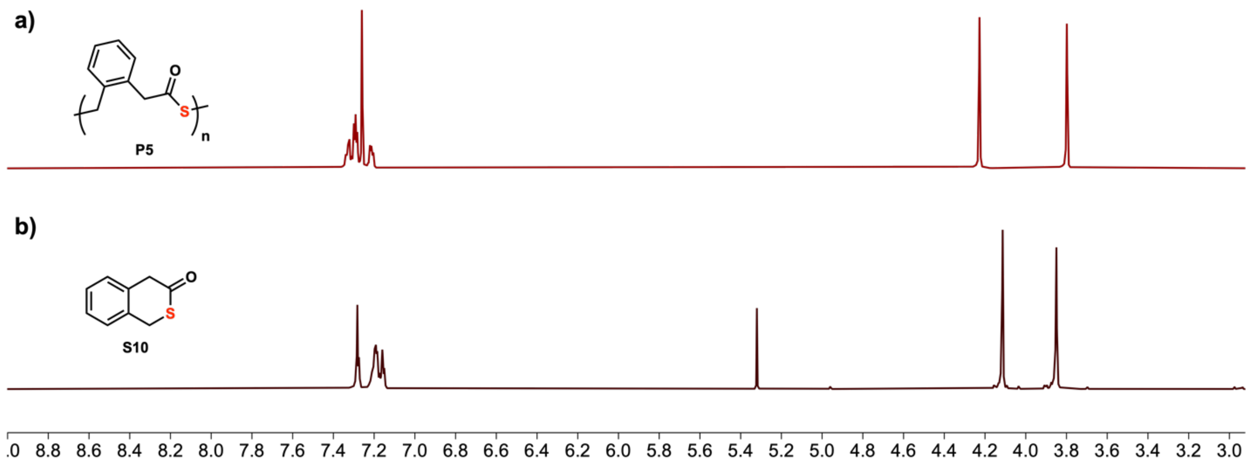

Figure S17. Comparison of **P5** before and after depolymerization. (a) ^{1}H NMR ($CDCl_3$) of **P5** (b) ^{1}H NMR ($CDCl_3$) of thiolactone product **S10**. Note the residual DCM at 5.30ppm in the spectra of **S10**.

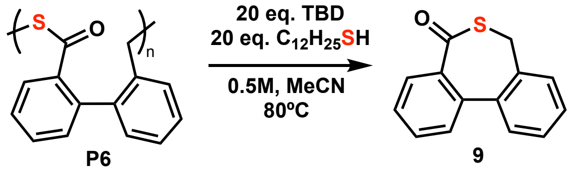

DEPOLYMERIZATION OF POLYMER P6: TBD (23.8mg, 0.1714mmol, 20 eq. relative to relative to the number of moles of polymer chain ends based on SEC measured M_n) was added to a vial containing **P6** (M_n = 35,003, DP = 155, D = 1.168) (300mg, 0.00857mmol, 1 eq.). The vial was placed under nitrogen before MeCN (2.66mL, volume chosen to produce a theoretical 0.5M concentration of thiolactone product), and dodecanethiol (41µL, 34.7mg, 20 eq.) were added. The reaction was heated to 80ºC and allowed to react for 2h. The reaction was removed from heat and allowed to cool for several minutes before TFA (20µL) was added. Crude ^{1}H NMR indicated that the reaction proceeded to full conversion. The crude product was then purified via flash chromatography (10% EtOAc, 90% hexanes) and further purified via recrystallization. To recrystallize, 10:1 hexanes: EtOAc was added to the product at 73ºC until the thiolactone dissolved. The solution was allowed to cool to room temperature at which point a solid precipitated. The solid was collected via filtration and was then triturated with cold hexanes to yield the product **9** (148mg, 49% yield). ^{1}H NMR (700 MHz, $CDCl_3$) δ 7.71 (ddd, *J* = 7.7, 1.4, 0.5 Hz, 1H), 7.62 (td, *J* = 7.6, 1.4 Hz, 1H), 7.50 (td, *J* = 7.6, 1.3 Hz, 1H), 7.43 – 7.35 (m, 4H), 7.34 – 7.30 (m, 1H), 4.29 (d, *J* = 14.1 Hz, 1H), 3.58 (d, *J* = 14.2 Hz, 1H).

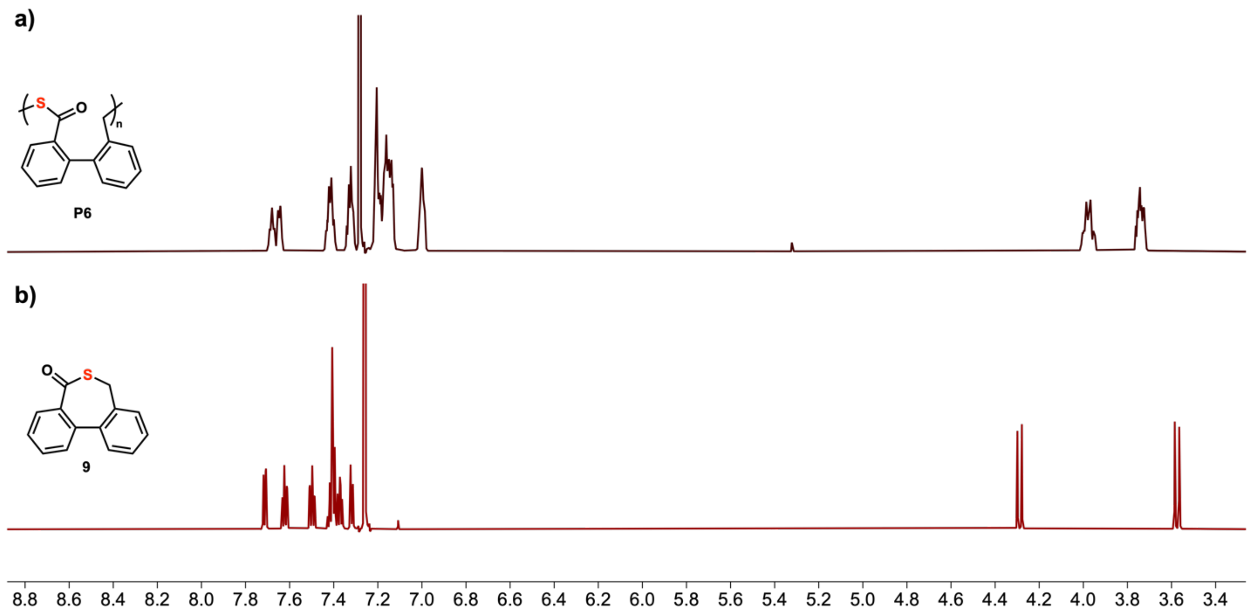

Figure S18. Comparison of P6 before and after depolymerization. (a) ^1H NMR ($CDCl_3$) of **P6** (b) ^1H NMR ($CDCl_3$) of thiolactone product **9**.

NMR CHARACTERIZATION OF POLYMERS

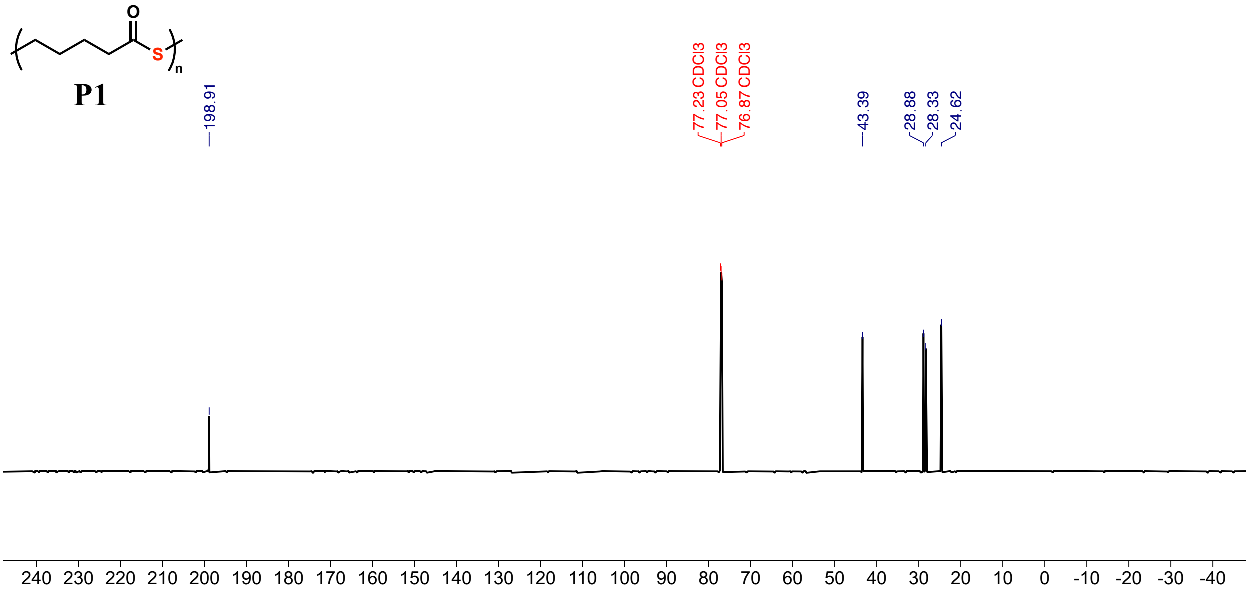

Figure S19. ^{13}C NMR ($CDCl_3$) of the precipitated polythiovalerolactone **P1**. ^{13}C NMR (176 MHz, $CDCl_3$) δ 198.91, 77.23, 77.05, 76.87, 43.39, 28.88, 28.33, 24.62.

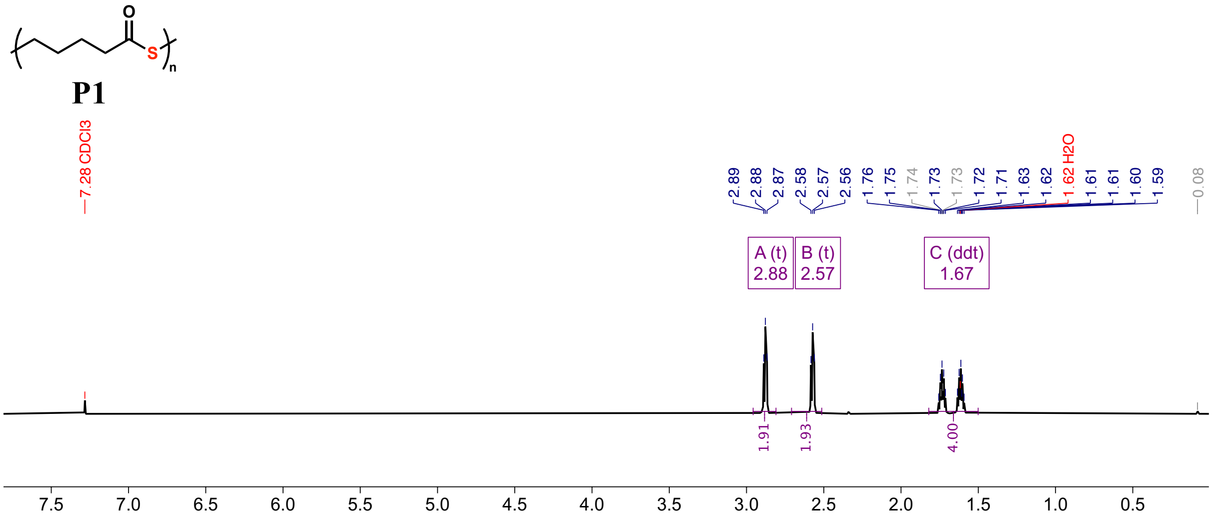

Figure S20. ^{1}H NMR ($CDCl_3$) of the precipitated polythiovalerolactone **P1**. ^{1}H NMR (700 MHz, $CDCl_3$) δ 2.88 (t, J = 7.3 Hz, 2H), 2.57 (t, J = 7.5 Hz, 2H), 1.67 (ddt, J = 84.5, 14.6, 7.2 Hz, 4H).

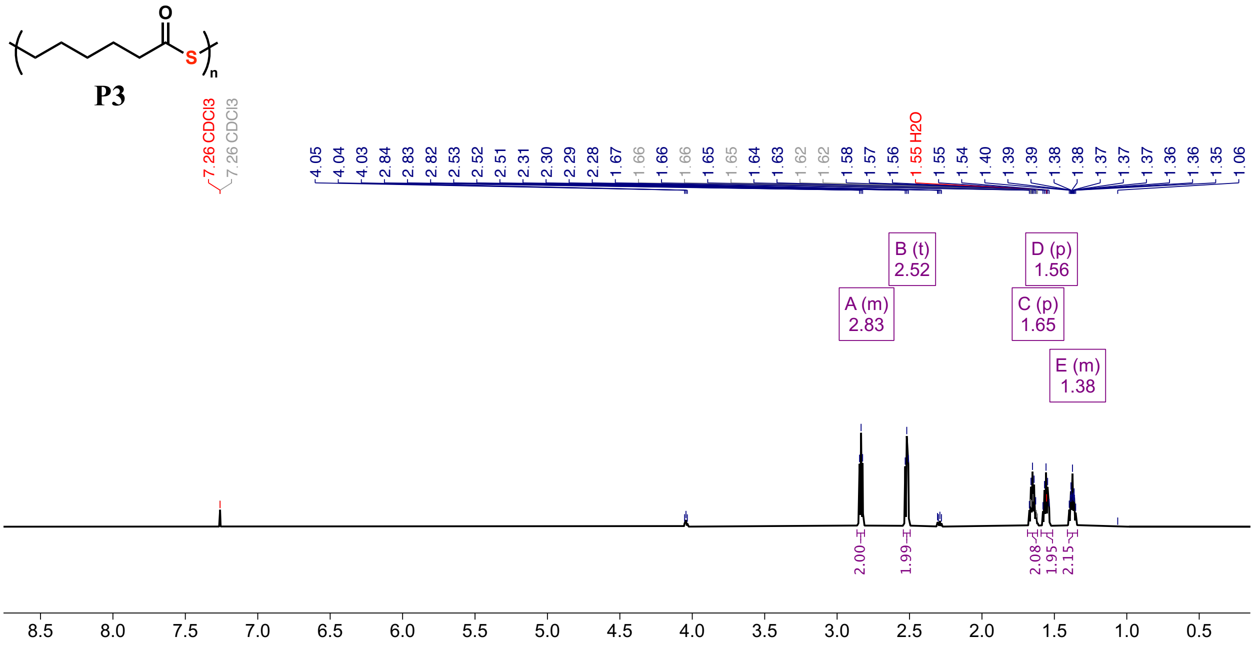

Figure S21. ^{1}H NMR ($CDCl_3$) of the precipitated polythiocaprolactone **P3**. ^{1}H NMR (700 MHz, CDCl3) δ 2.86 – 2.81 (m, 2H), 2.52 (t, J = 7.5 Hz, 2H), 1.65 (p, J = 7.6 Hz, 2H), 1.56 (p, J = 7.4 Hz, 2H), 1.41 – 1.34 (m, 2H).

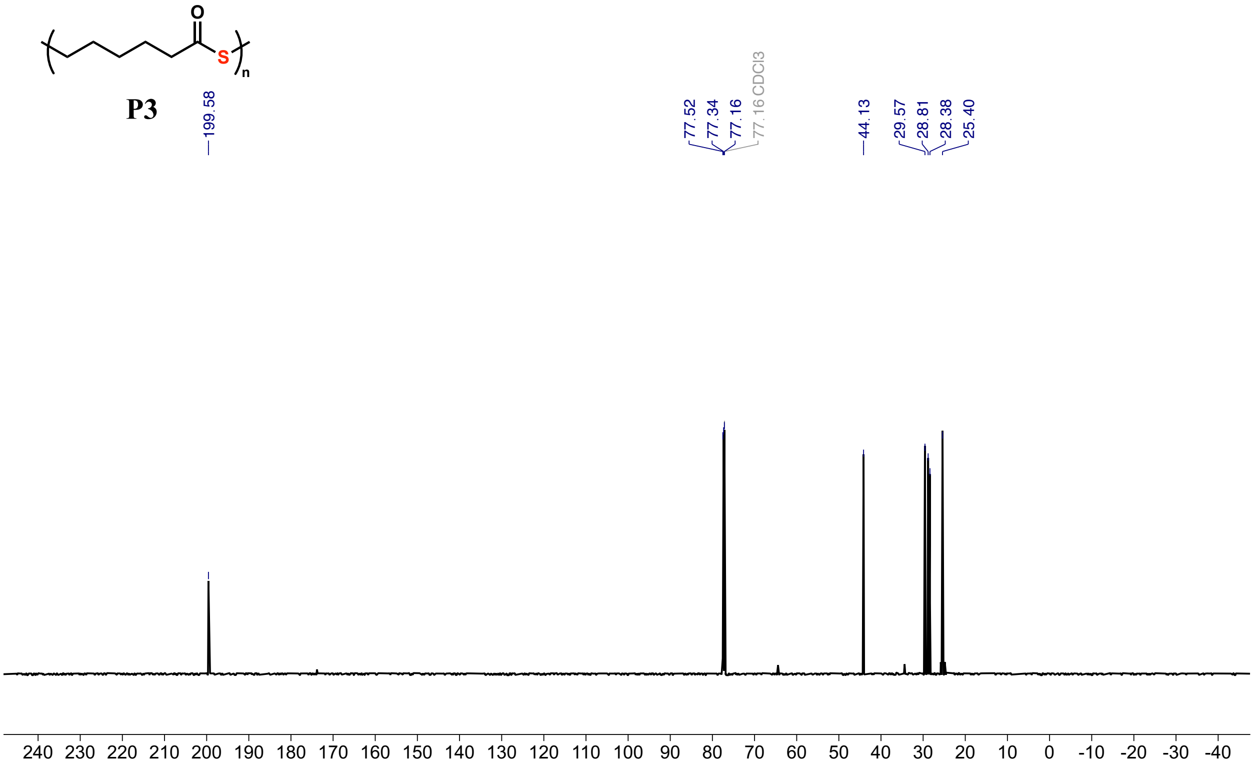

Figure S22. ^{13}C NMR ($CDCl_3$) of precipitated polythiocaprolactone **P3**. ^{13}C NMR (176 MHz, $CDCl_3$) δ 199.58, 77.52, 77.34, 77.16, 44.13, 29.57, 28.81, 28.38, 25.40.

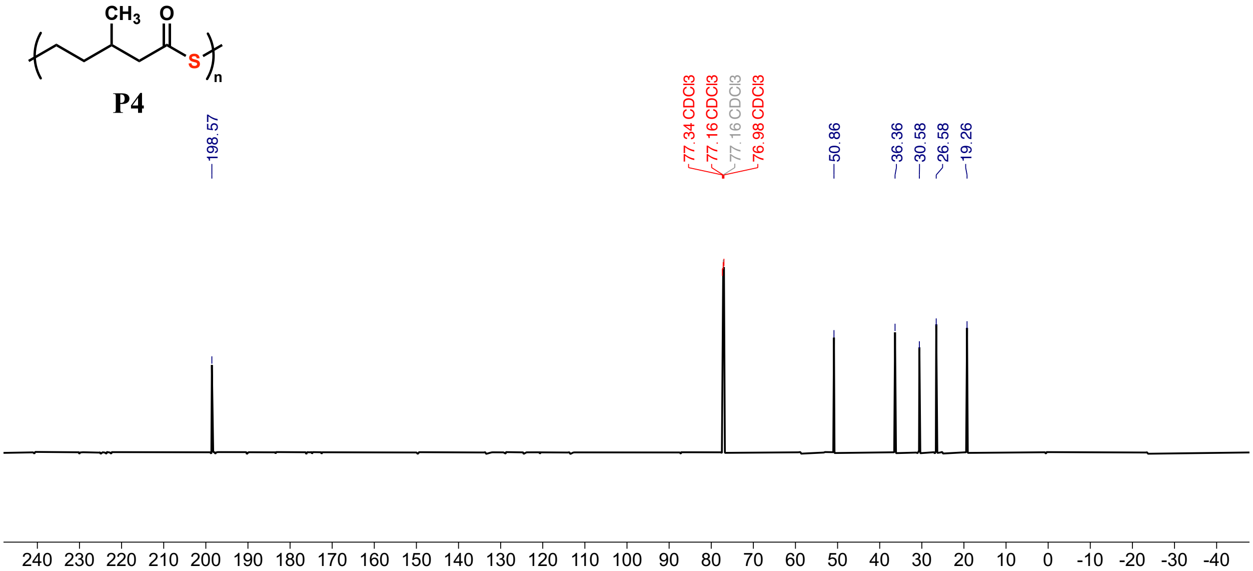

Figure S23. ^{13}C NMR ($CDCl_3$) of precipitated polybeta-methylvalerolactone **P4**. ^{13}C NMR (176 MHz, $CDCl_3$) δ 198.57, 77.34, 77.16, 76.98, 50.86, 36.36, 30.58, 26.58, 19.26.

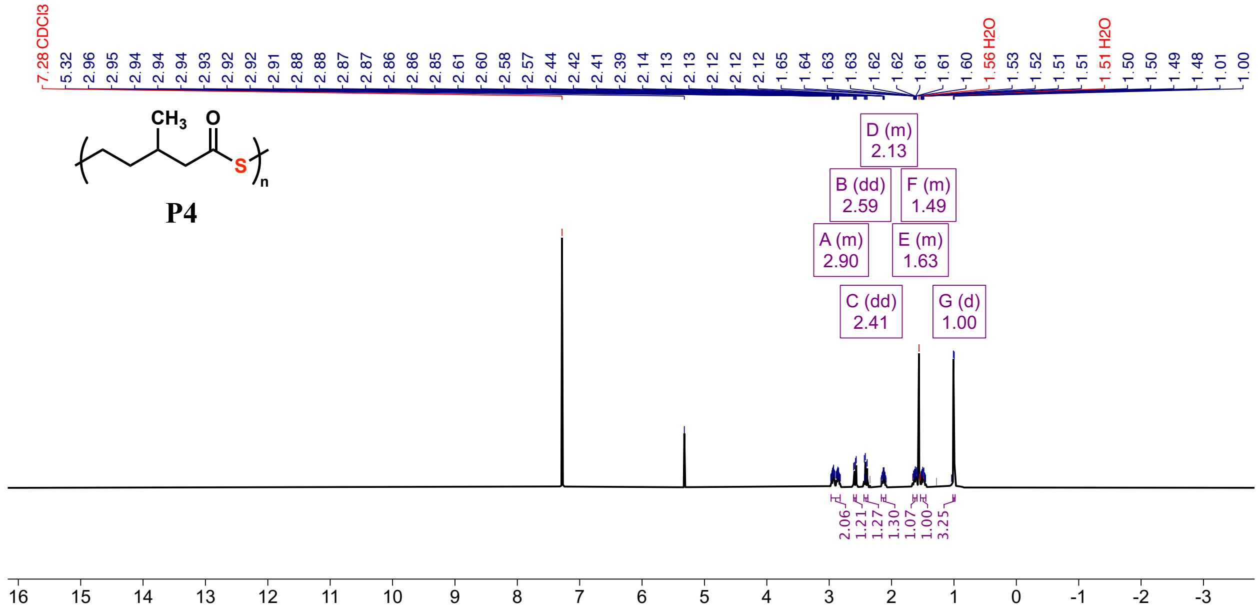

Figure S24. ^{1}H NMR ($CDCl_3$) of precipitated polybeta-methylvalerolactone **P4**. ^{1}H NMR (500 MHz, $CDCl_3$) δ 2.97 – 2.82 (m, 2H), 2.59 (dd, J = 14.6, 5.8 Hz, 1H), 2.41 (dd, J = 14.7, 8.2 Hz, 1H), 2.16 – 2.09 (m, 1H), 1.66 – 1.59 (m, 1H), 1.54 – 1.45 (m, 1H), 1.00 (d, J = 6.7 Hz, 3H). Note the residual dichloromethane at 5.32ppm from precipitation.

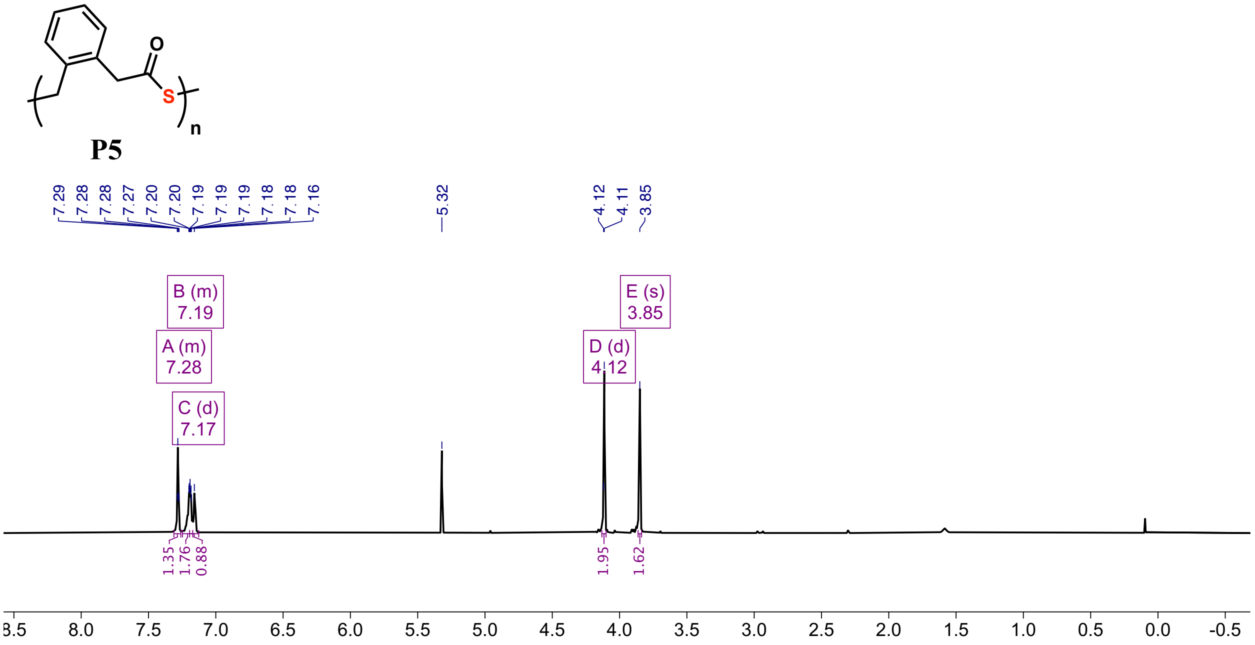

Figure S25. ^{1}H NMR ($CDCl_3$) of precipitated polythiochromanone **P5**. ^{1}H NMR (700 MHz, $CDCl_3$) δ 7.31 – 7.26 (m, 1H), 7.25 – 7.17 (m, 2H), 7.17 (d, J = 15.5 Hz, 1H), 4.12 (d, J = 3.5 Hz, 2H), 3.85 (s, 2H). Note the residual dichloromethane at 5.32ppm from precipitation.

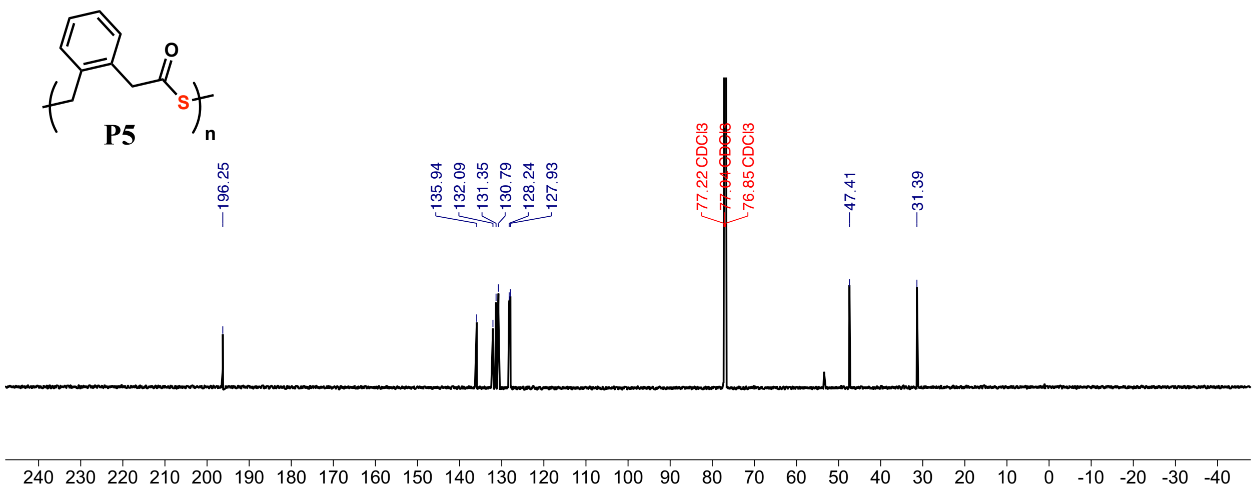

Figure S26. ^{13}C NMR ($CDCl_3$) of precipitated polythiochromanone **P5**. ^{13}C NMR (176 MHz, $CDCl_3$) δ 196.25, 135.94, 132.09, 131.35, 130.79, 128.24, 127.93, 77.22, 77.04, 76.85, 47.41, 31.39.

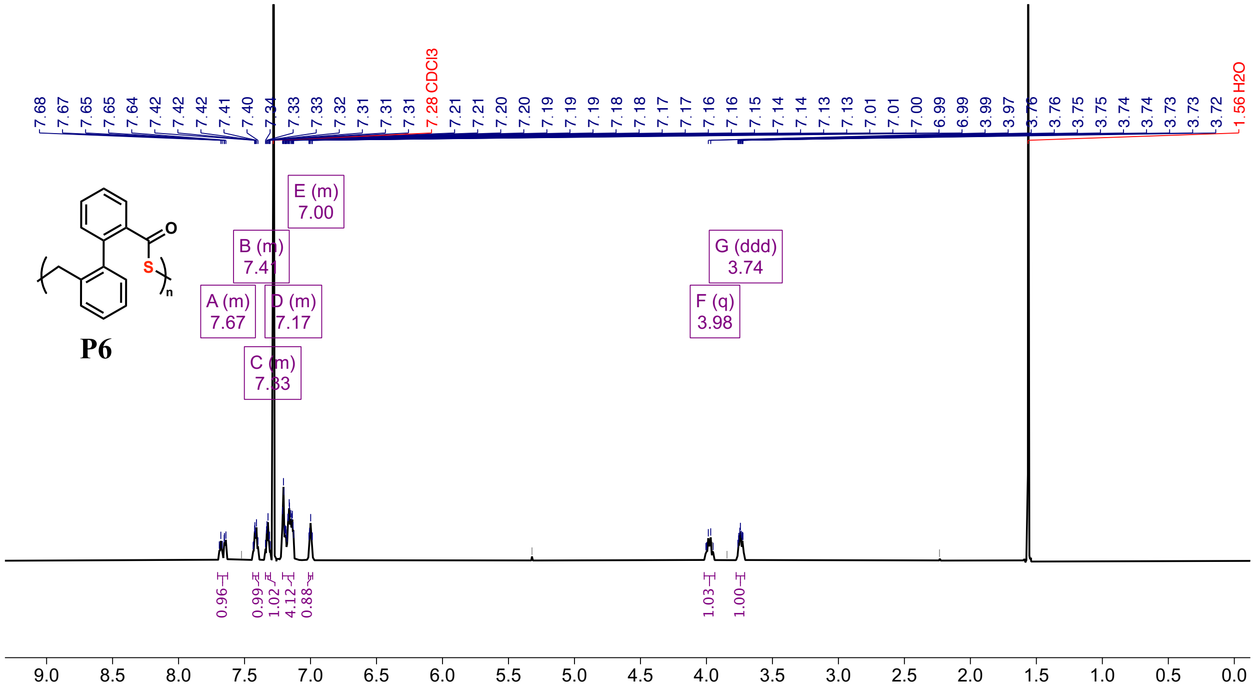

Figure S27. ^{1}H NMR ($CDCl_3$) of the precipitated **P6**. ^{1}H NMR (700 MHz, $CDCl_3$) δ 7.70 – 7.63 (m, 1H), 7.44 – 7.39 (m, 1H), 7.34 – 7.30 (m, 1H), 7.21 – 7.13 (m, 4H), 7.02 – 6.98 (m, 1H), 3.98 (q, *J* = 12.8 Hz, 1H), 3.74 (ddd, *J* = 13.5, 6.9, 3.2 Hz, 1H).

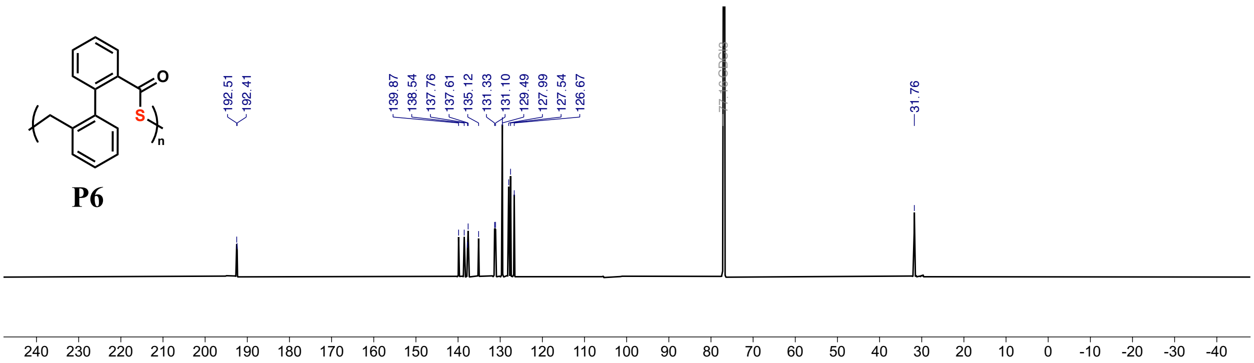

Figure S28. ^{13}C NMR ($CDCl_3$) of the precipitated **P6**. ^{13}C NMR (176 MHz, $CDCl_3$) δ 192.51, 192.41, 139.87, 138.54, 137.76, 137.61, 135.12, 131.33, 131.10, 129.49, 127.99, 127.54, 126.67, 31.76.

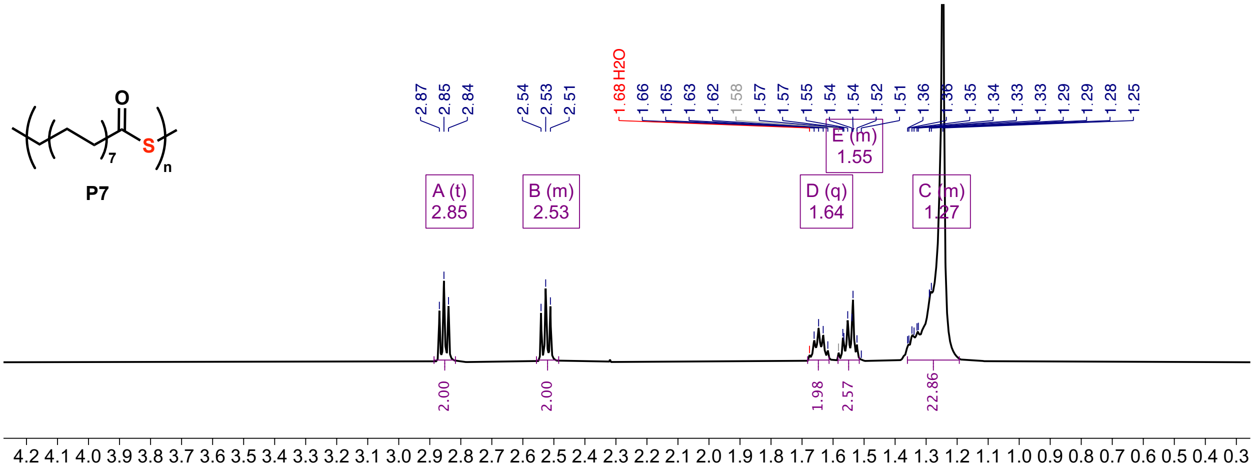

Figure S29. ^{1}H NMR ($CDCl_3$) of precipitated polythiohexadecanolide **P7**. ^{1}H NMR (500 MHz, $CDCl_3$) δ 2.85 (t, J = 7.4 Hz, 2H), 2.56 – 2.48 (m, 2H), 1.64 (q, J = 7.3 Hz, 2H), 1.57 – 1.52 (m, 2H), 1.25 (s, 22H).

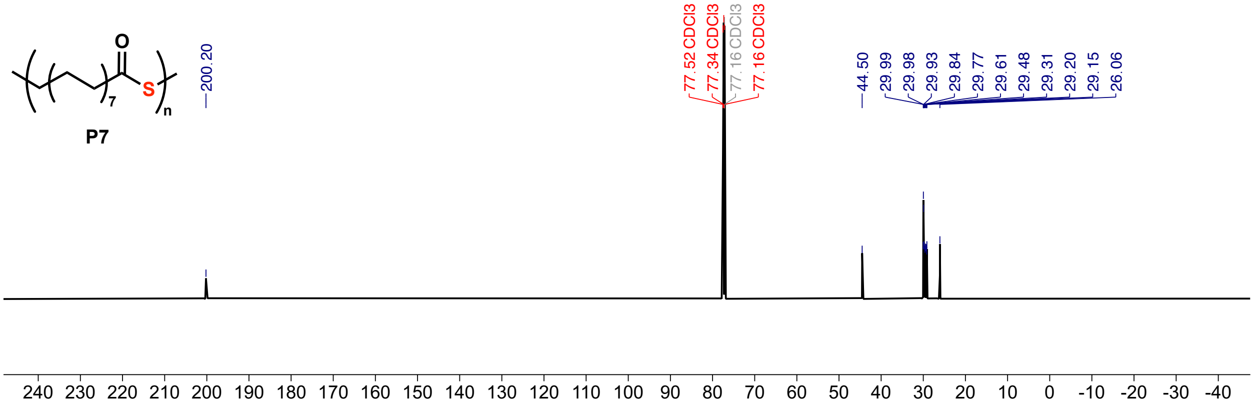

Figure S30. ^{13}C NMR ($CDCl_3$) of precipitated polythiohexadecanolide **P7**. ^{13}C NMR (176 MHz, $CDCl_3$) δ 200.20, 77.52, 77.34, 77.16, 44.50, 29.99, 29.98, 29.93, 29.84, 29.77, 29.61, 29.48, 29.31, 29.20, 29.15, 26.06.

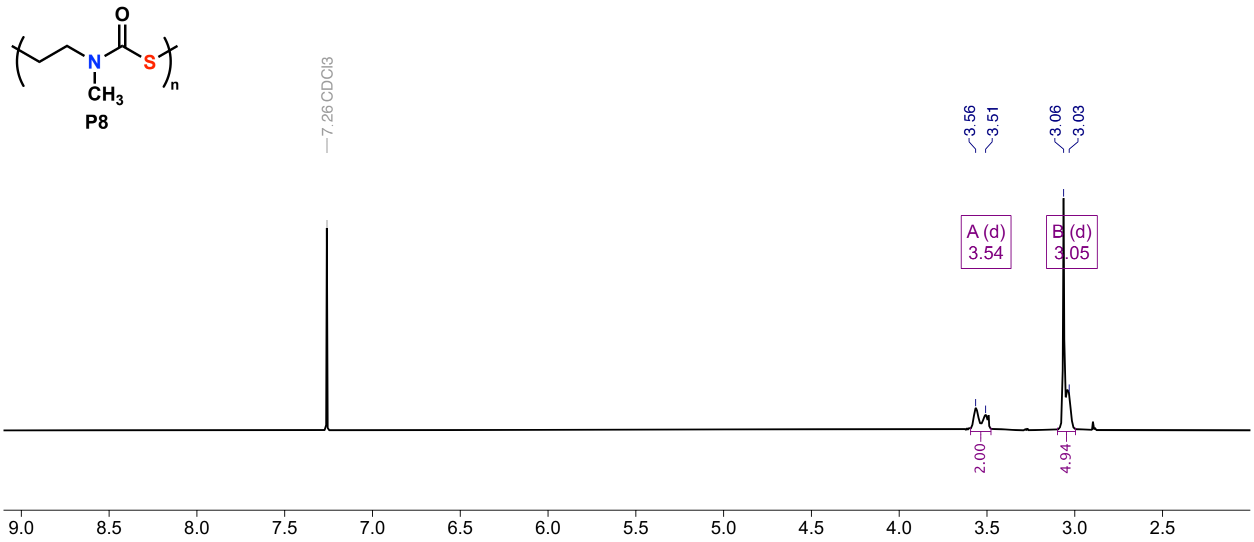

Figure S31. ^{1}H NMR ($CDCl_3$) of precipitated poly-3-methyloxazolidine-2-thione **P8**. ^{1}H NMR (700 MHz, $CDCl_3$) δ 3.54 (d, J = 39.8 Hz, 2H), 3.05 (d, J = 22.3 Hz, 5H).

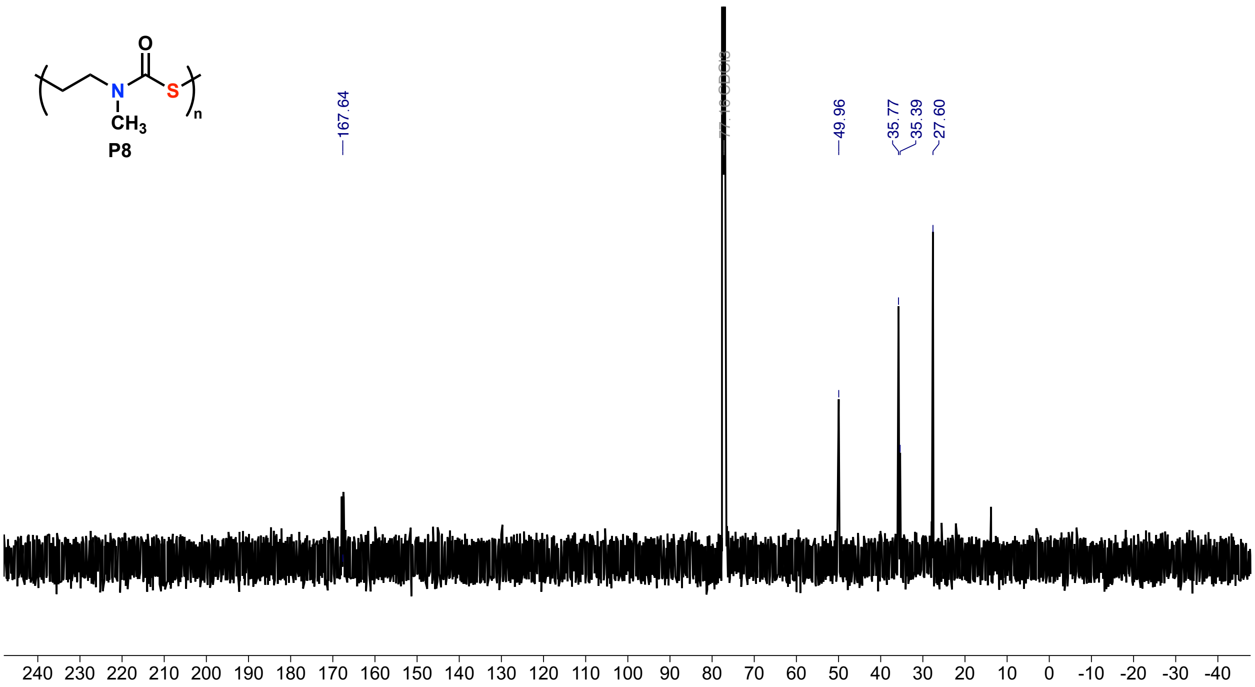

Figure S32. ^{13}C NMR ($CDCl_3$) of precipitated poly-3-methyloxazolidine-2-thione **P8**. Polymer was poorly soluble in $CDCl_3$ and other common deuterated solvents. ^{13}C NMR (176 MHz, $CDCl_3$) δ 167.64, 49.96, 35.77, 35.39, 27.60.

SEC CHARACTERIZATION

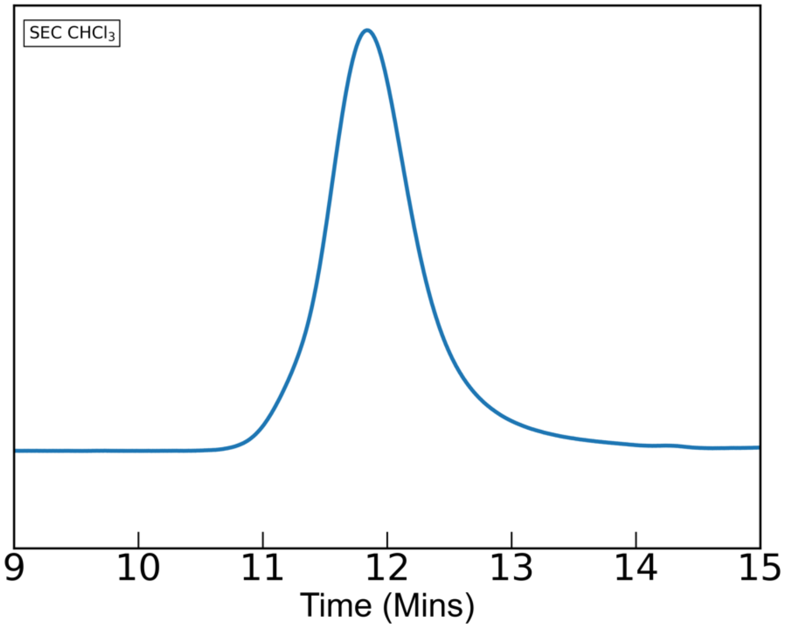

Figure S33. SEC trace of polymerization of thionovalerolactone monomer **1** to polymer **P1** at a target DP of 200. M_n = 14,010 g/mol; M_w = 21,470 g/mol; Đ = 1.533.

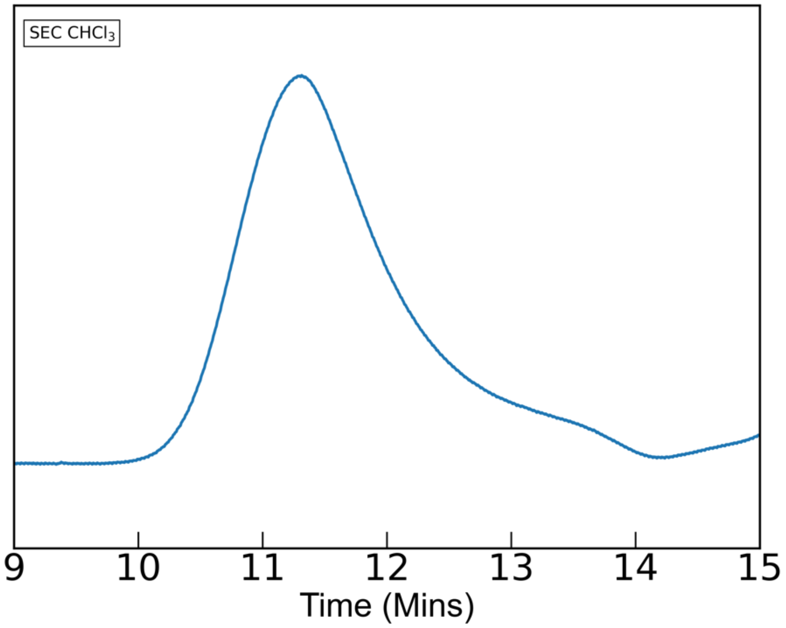

Figure S34. SEC trace of polymerization of thionobutyrolactone monomer **2** to polymer **P2** at a target DP of 200. M_n = 23,742 g/mol; M_w=42,473 g/mol; Đ = 1.789.

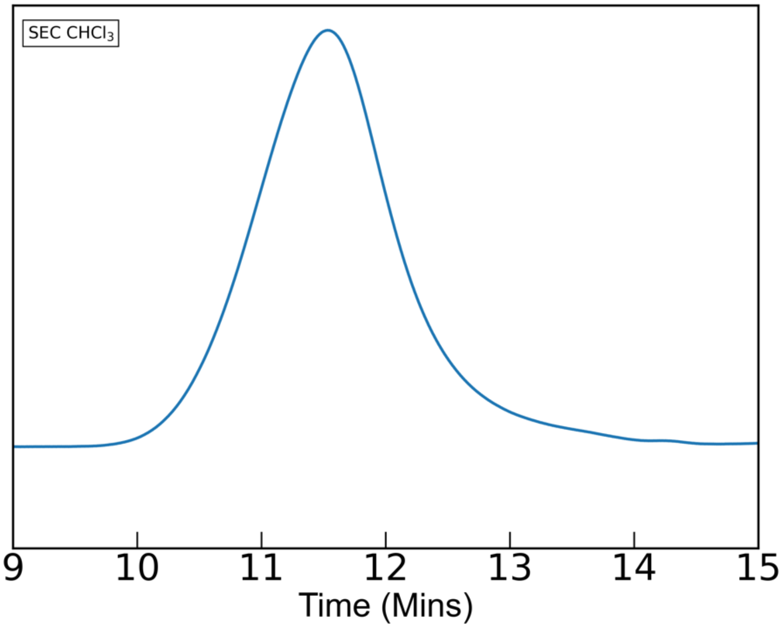

Figure S35. SEC trace of polymerization of thionocaprolactone monomer **3** to polymer **P3** at a target DP of 200. M_n = 19,068 g/mol; M_w = 41,105 g/mol; D =2.156

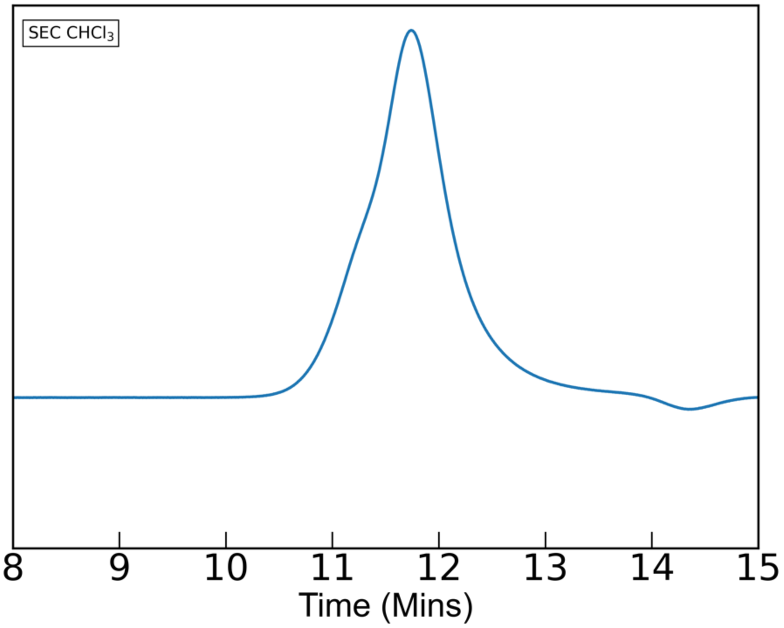

Figure S36. SEC trace of polymerization of beta-methyl thionovalerolactone monomer **4** to polymer **P4** a target DP of 200. M_n = 21,629g/mol; M_w = 32,667 g/mol; D = 1.510

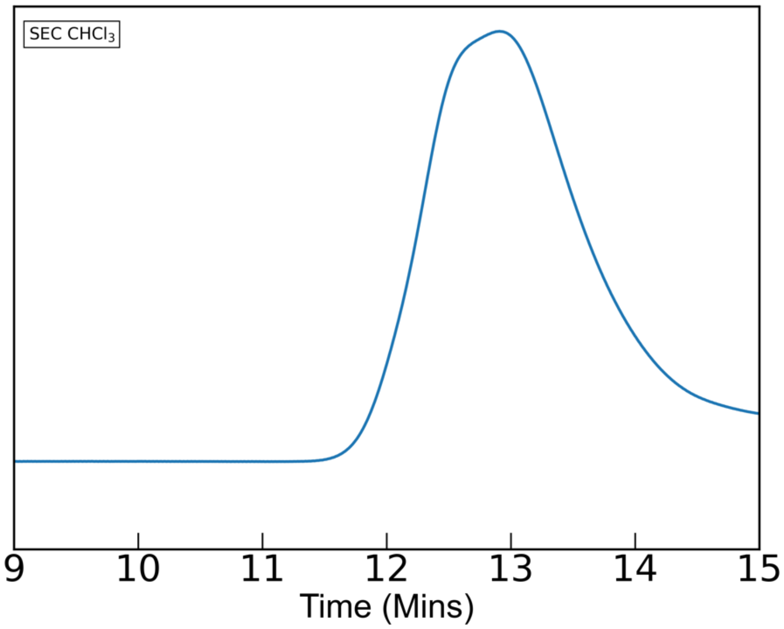

Figure S37. SEC trace of polymerization of thionochromanone monomer **5** to polymer **P5** at a target DP of 200. M_n = 2,871 g/mol; M_w = 5,758 g/mol; D = 2.006

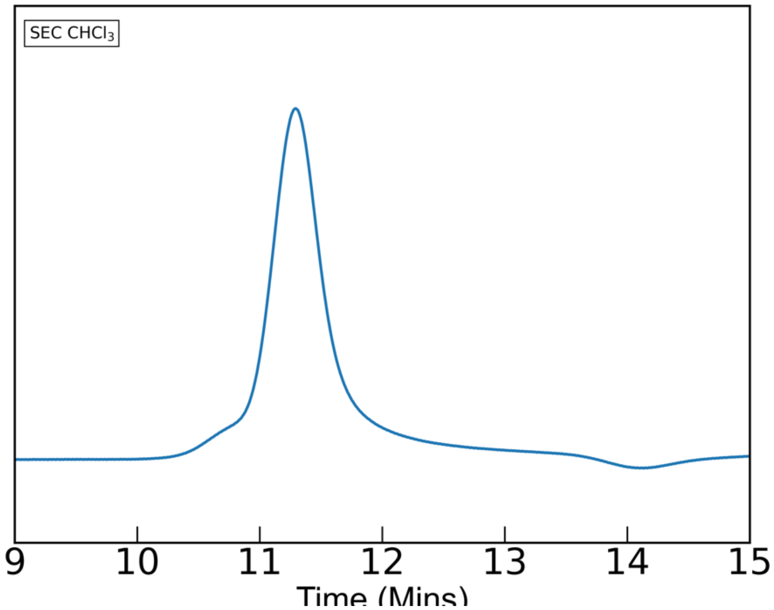

Figure S38. SEC trace of monomer **6** to polymer **P6** at a target DP of 200. M_n = 35,003g/mol; M_w = 40,900g/mol; D = 1.168.

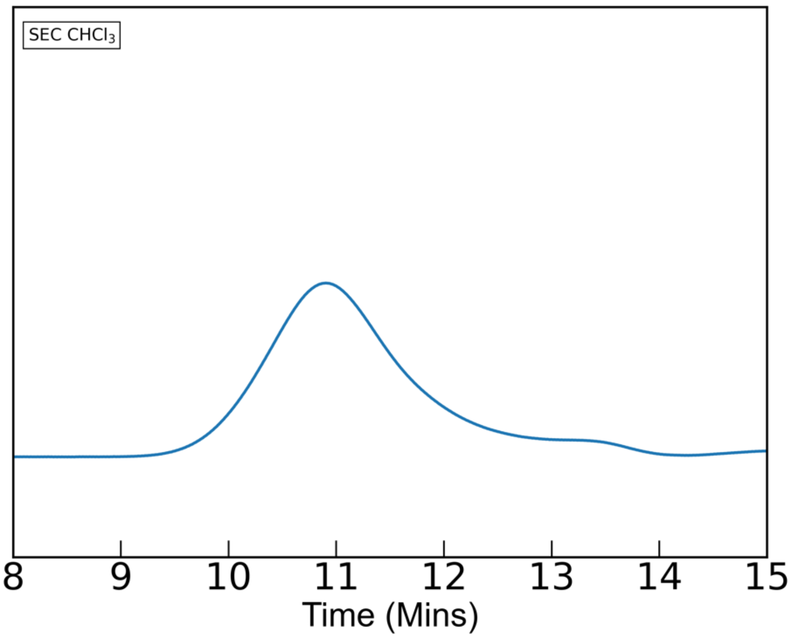

Figure S39. SEC trace of macrocyclic monomer **7** to polymer **P7** a target DP of 200. Molecular weight characterization performed via Multi Angle Laser Light Scattering (MALLS): M_n = 36,710 g/mol; M_w = 51,570g/mol; Đ = M_w/M_n = 1.405.

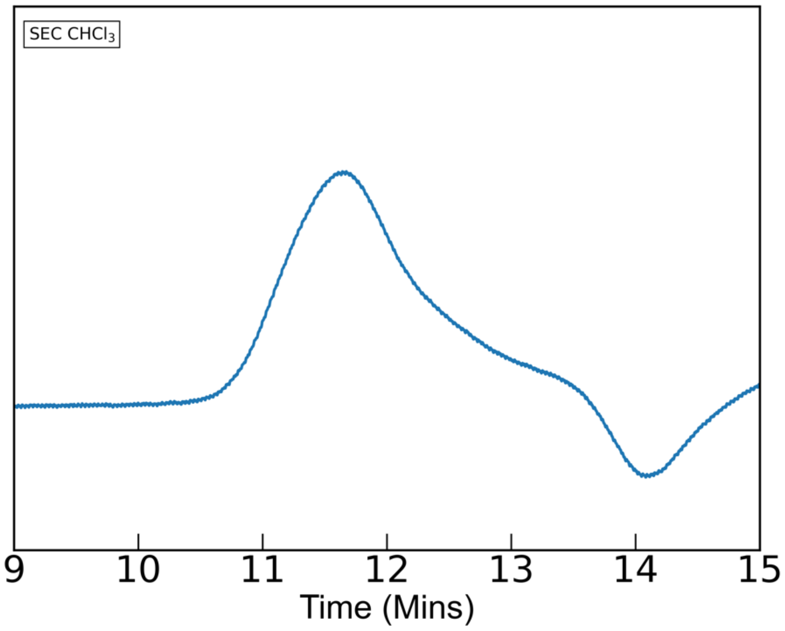

Figure S40. SEC trace of thionocarbamate monomer **8** to polymer **P8** a target DP of 200. M_n = 20,595 g/mol; M_w = 29,322 g/mol; Đ = 1.424

TGA

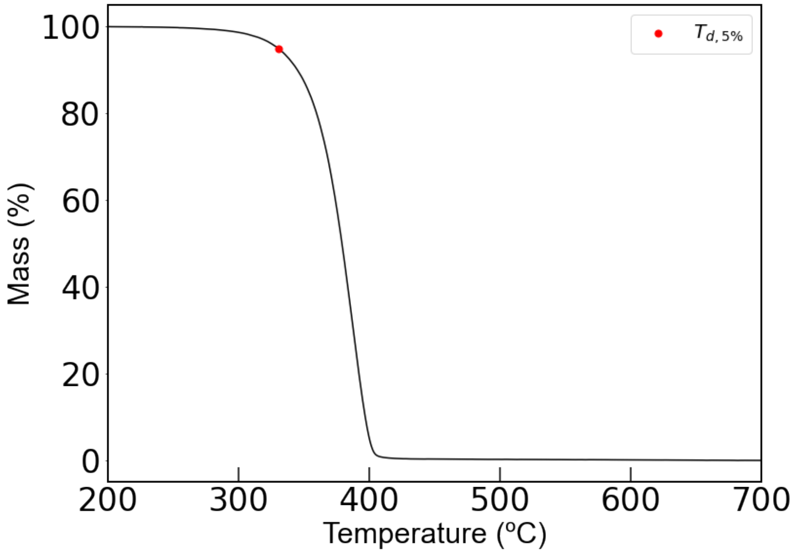

Figure S41. TGA of the beta-methyl polythioester **P4**. $T_{d,5\%}$ = 330ºC

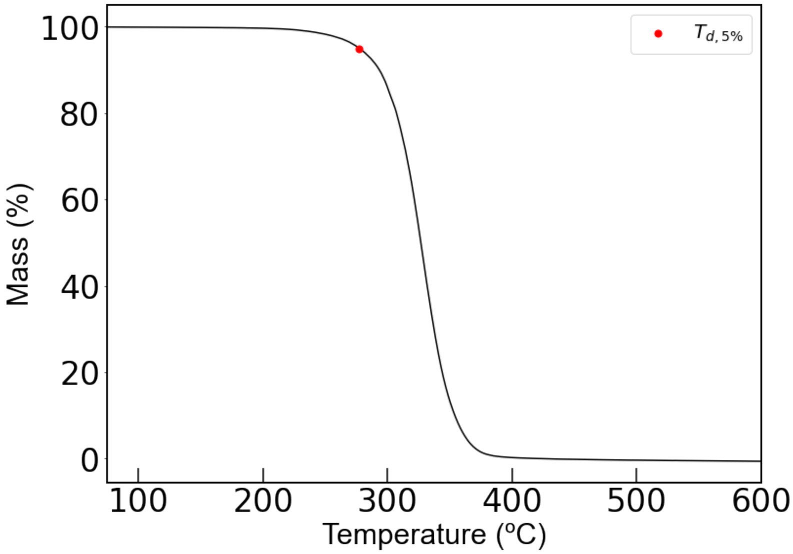

Figure S42. TGA of polythiochromanone **P5.** $T_{d,5\%}$ = 278ºC

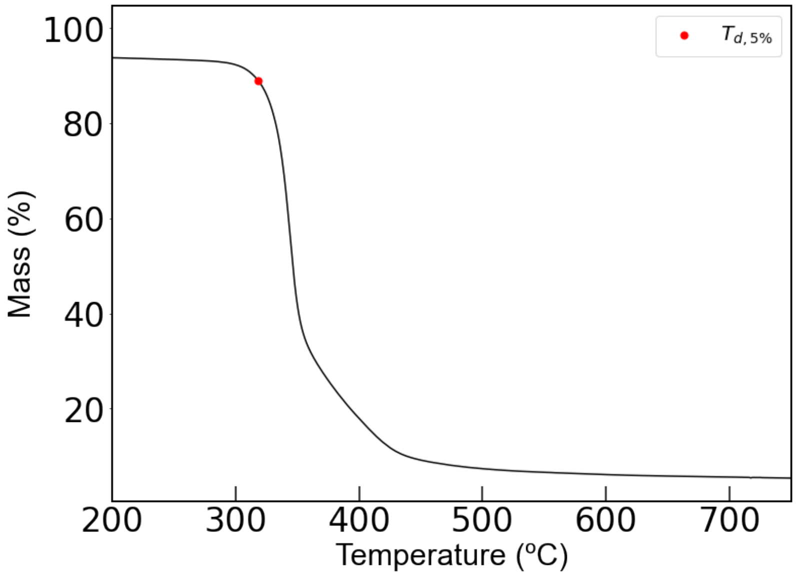

Figure S43. TGA of the polymer **P6**. $T_{d,5\%}$ = 318ºC

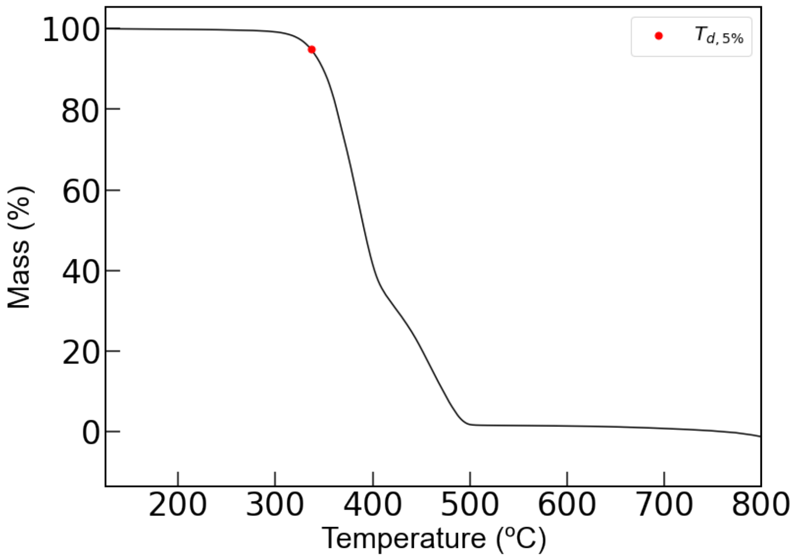

Figure S44. TGA of the macrocyclic polymer **P7**. $T_{d,5\%}$ = 336ºC

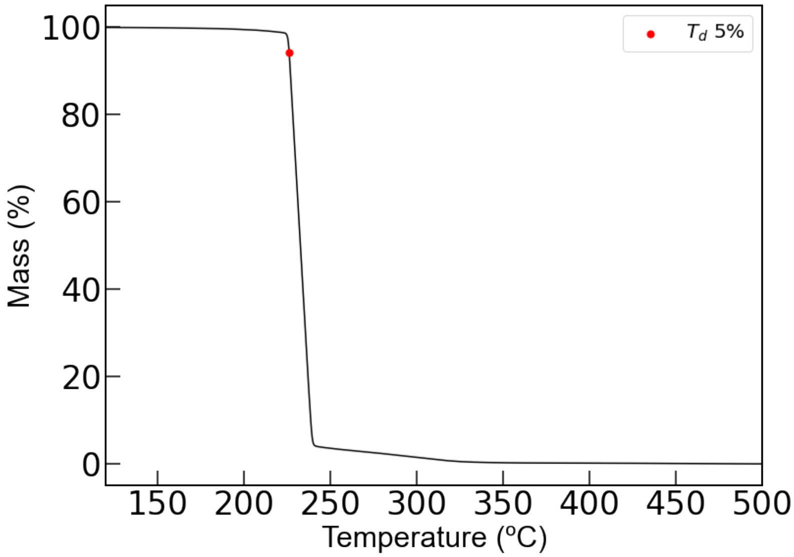

Figure S45. TGA of the polythiocarbamate **P8**.$T_{d,5\%}$ = 226ºC

DSC

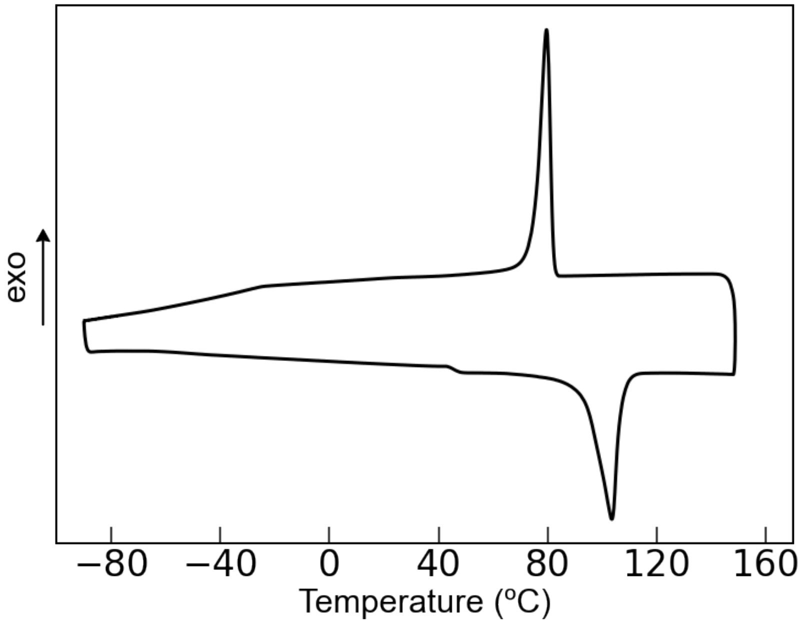

Figure S46. DSC curve of polythiocaprolactone **P3**. T_g = 45ºC, T_m = 103ºC , and T_{cryst} = 85ºC

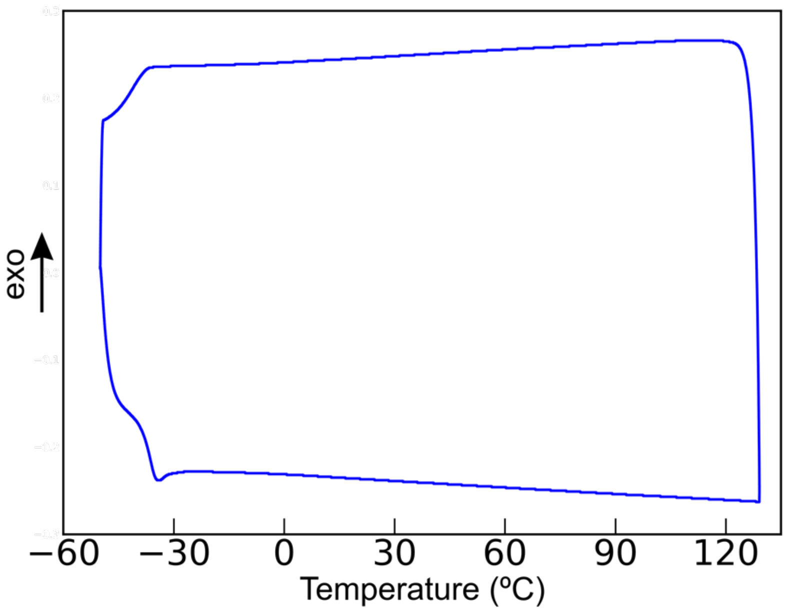

Figure S47. DSC of polymer **P4**. T_g = -36ºC

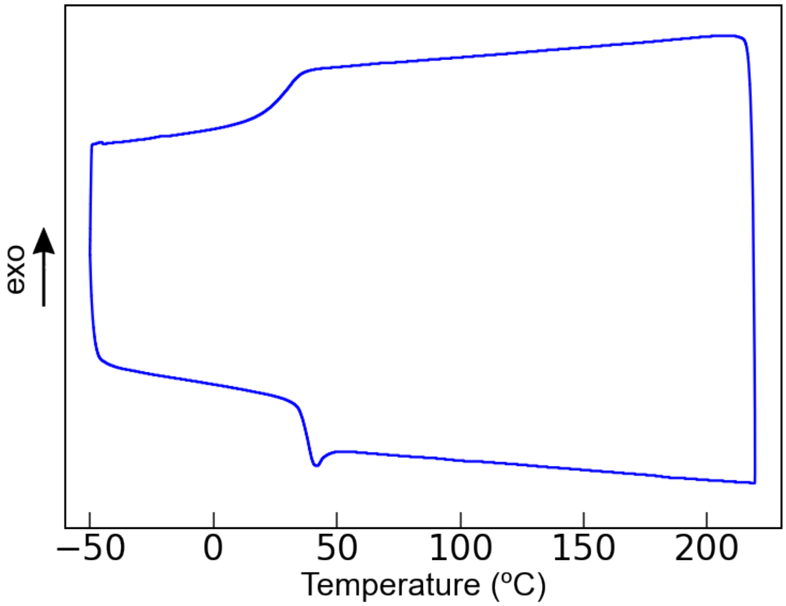

Figure S48. DSC of polythiochromanone **P5**. T_g = 39ºC

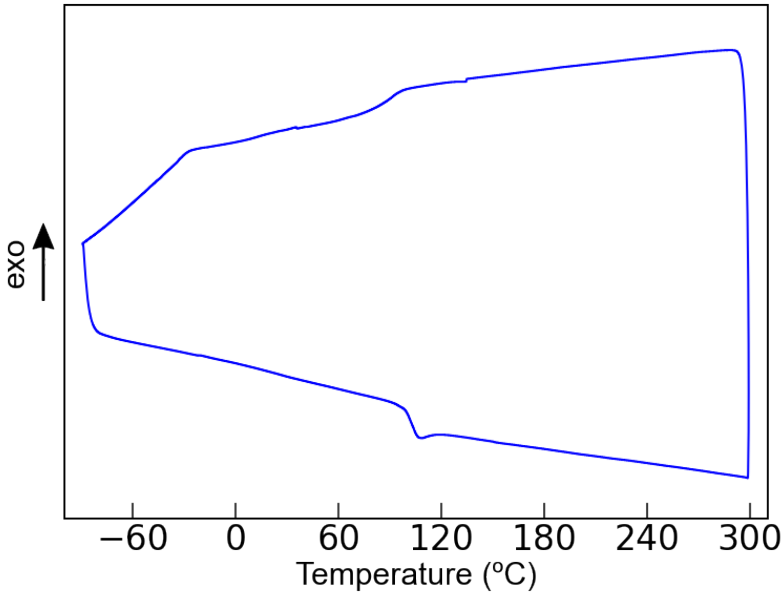

Figure S49. DSC of polymer **P6**, T_g = 104ºC.

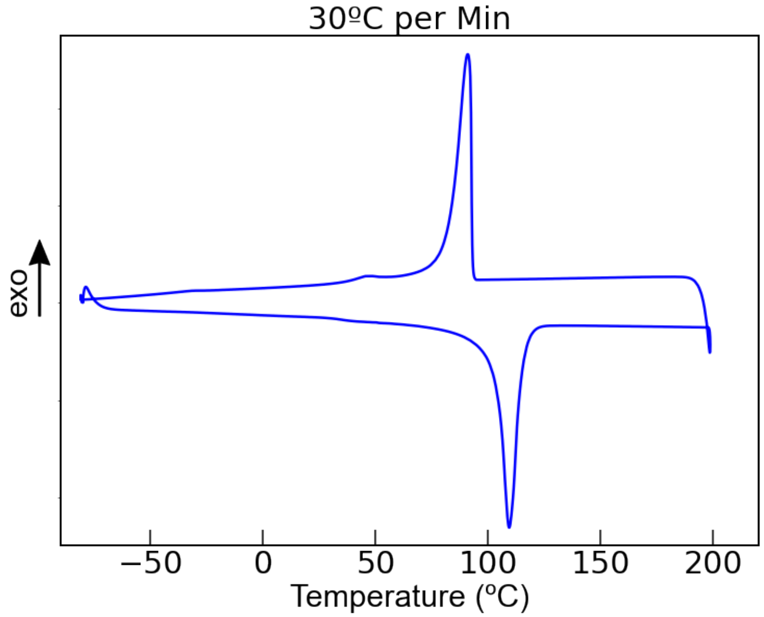

Figure S50. DSC of macrocylic polymer **P7** at a ramp heating and cooling rate of 30ºC/min. T_g < -70ºC (not observed), T_m= 110ºC, T_{cryst} = 91ºC.

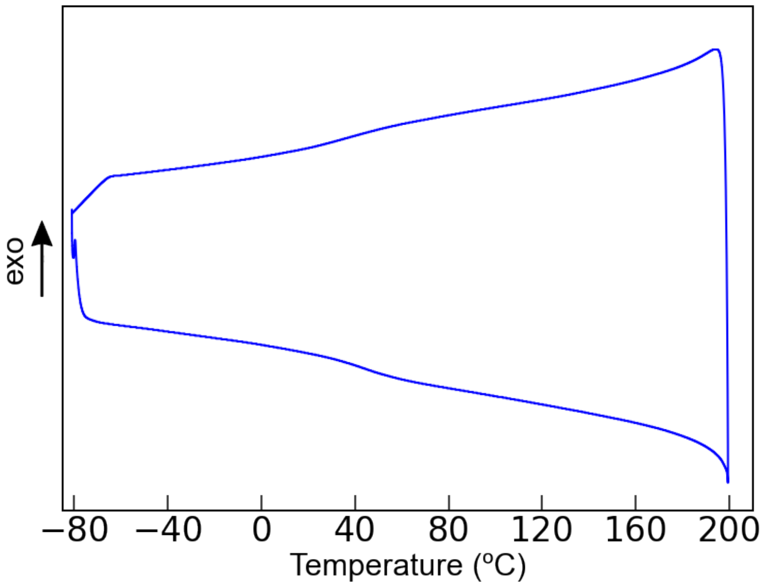

Figure S51. DSC of polythiocarbamate **P8**. T_g = 43ºC

COMPUTATIONAL STUDIES

Structures were preliminarily minimized via molecular mechanics (Generalized Amber Force Field GAFF) using the Avogadro software package.[19,20] Molecular mechanics optimized coordinates were then further optimized using Psi4 with the standard grid size (75,302).[21] Geometry optimizations and frequency analysis were performed at the M06-2x/6-311++G** level of theory. All optimized geometries were verified as minima via the absence of imaginary vibrational frequencies. Orbital visualization was performed using VMD.[22]

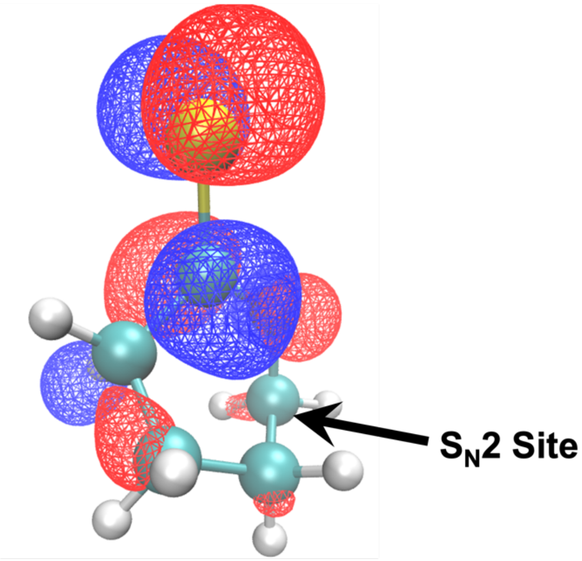

Figure S52. DFT optimized geometry and LUMO visualization of monomer **1** at an isolevel of +/- 0.035.

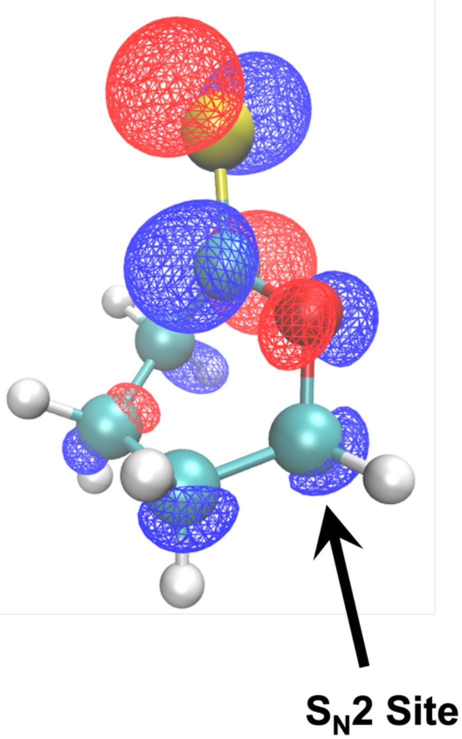

Figure S53. LUMO+1 visualization of monomer **1** at an isolevel of +/- 0.035 with electrophilic S_N2 site highlighted.

DFT Optimized Geometry of thionovalerolactone 1

S	-2.196989855216	0.049793175401	-0.061439032038
C	-0.591198632704	-0.009133769791	0.209674386349
O	0.060632613209	-1.172459653693	0.138187690385
C	1.489761979921	-1.147583646230	0.282571994314
C	2.080081332126	-0.124624301868	-0.673269237502
C	1.492039951609	1.272243198064	-0.387631731446
C	0.272534254252	1.176479968113	0.551155733670
H	1.809981952019	-2.162037643048	0.055095838192
H	1.743660125169	-0.927119510225	1.324165549825
H	1.834024787224	-0.440423854900	-1.689423652442
H	3.168060603140	-0.129354864580	-0.585090363207
H	1.184698105391	1.746385122390	-1.320629195521
H	2.236140466048	1.924044714909	0.073089247026

H	-0.334341330115	2.077032848063	0.523399044891
H	0.615732308907	1.039817520492	1.583691948674

KINETIC STUDIES

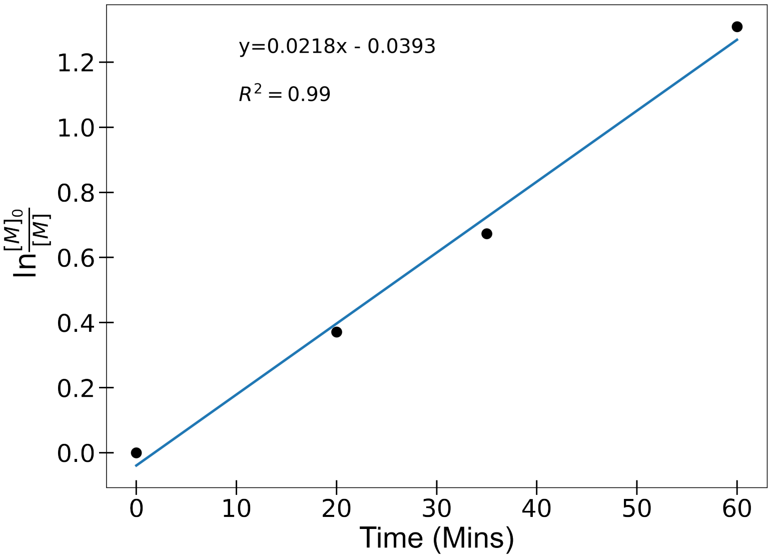

Figure S54. Kinetic studies of monomer **1** polymerized with 0.01 eq. tetrabutylammonium thioacetate at 5M in DMA.

REFERENCES

(1) Curphey, T. J. Thionation with the Reagent Combination of Phosphorus Pentasulfide and Hexamethyldisiloxane. *J. Org. Chem.* **2002**, *67* (18), 6461–6473. https://doi.org/10.1021/jo0256742.
(2) Yuan, P.; Sun, Y.; Xu, X.; Luo, Y.; Hong, M. Towards High-Performance Sustainable Polymers via Isomerization-Driven Irreversible Ring-Opening Polymerization of Five-Membered Thionolactones. *Nat. Chem.* **2022**, *14* (3), 294–303. https://doi.org/10.1038/s41557-021-00817-9.
(3) Prebihalo, E. A.; Luke, A. M.; Reddi, Y.; LaSalle, C. J.; Shah, V. M.; Cramer, C. J.; Reineke, T. M. Radical Ring-Opening Polymerization of Sustainably-Derived Thionoisochromanone. *Chem. Sci.* **2023**, *14* (21), 5689–5698. https://doi.org/10.1039/D2SC06040J.
(4) Smith, R. A.; Fu, G.; McAteer, O.; Xu, M.; Gutekunst, W. R. Radical Approach to Thioester-Containing Polymers. *J. Am. Chem. Soc.* **2019**, *141* (4), 1446–1451. https://doi.org/10.1021/jacs.8b12154.
(5) M. Bingham, N.; J. Roth, P. Degradable Vinyl Copolymers through Thiocarbonyl Addition–Ring-Opening (TARO) Polymerization. *Chemical Communications* **2019**, *55* (1), 55–58. https://doi.org/10.1039/C8CC08287A.
(6) Zaorska, E.; Gawryś-Kopczyńska, M.; Ostaszewski, R.; Ufnal, M.; Koszelewski, D. Evaluation of Thionolactones as a New Type of Hydrogen Sulfide (H2S) Donors for a Blood Pressure Regulation. *Bioorganic Chemistry* **2021**, *108*, 104650. https://doi.org/10.1016/j.bioorg.2021.104650.

(7) Hicken, E. J.; Corey, E. J. Stereoselective Synthesis of Woody Fragrances Related to Georgyone and Arborone. *Org. Lett.* **2008**, *10* (6), 1135–1138. https://doi.org/10.1021/ol8000359.
(8) Jacobi, P. A.; Egbertson, M.; Frechette, R. F.; Miao, C. K.; Weiss, K. T. Thiazoles in Organic Synthesis. Novel Syntheses of Menthanes and Eremophilanes. *Tetrahedron* **1988**, *44* (11), 3327–3338. https://doi.org/10.1016/S0040-4020(01)85966-4.
(9) Awheda, I.; Saygili, N.; Garner, A. C.; Wallis, J. D. Activation and Regioselectivity of Five-Membered Cyclic Thionocarbamates to Nucleophilic Attack. *RSC Adv.* **2013**, *3* (47), 24997–25009. https://doi.org/10.1039/C3RA41074A.
(10) Lown, W.J.; Chauhan, S.M.S. Stereoelectronic control in the aqueous decomposition of novel nitrosothioureas.*J. Org. Chem.* **1983**, 48(22), 3901-3908. https://doi.org/10.1021/jo00170a006.
(11) Datta, P. P.; Kiesewetter, M. K. Controlled Organocatalytic Ring-Opening Polymerization of ε-Thionocaprolactone. *Macromolecules* **2016**, *49* (3), 774–780. https://doi.org/10.1021/acs.macromol.6b00136.
(12) Xia, Y.; Yuan, P.; Zhang, Y.; Sun, Y.; Hong, M. Converting Non-Strained γ-Valerolactone and Derivatives into Sustainable Polythioesters via Isomerization-Driven Cationic Ring-Opening Polymerization of Thionolactone Intermediate. *Angewandte Chemie International Edition* **2023**, *62* (14), e202217812. https://doi.org/10.1002/anie.202217812.
(13) Hakimelahi, G. H.; Just, G. The Synthesis of 4-Decarboxy-4-Phosphono-O-2-Isooxacephems, -Isopenams and -Isooxacephems Containing Phosphorus at the 3-Position. *Helvetica Chimica Acta* **1982**, *65* (5), 1359–1367. https://doi.org/10.1002/hlca.19820650505.
(14) Stellmach, K. A.; Paul, M. K.; Xu, M.; Su, Y.-L.; Fu, L.; Toland, A. R.; Tran, H.; Chen, L.; Ramprasad, R.; Gutekunst, W. R. Modulating Polymerization Thermodynamics of Thiolactones Through Substituent and Heteroatom Incorporation. *ACS Macro Lett.* **2022**, *11* (7), 895–901. https://doi.org/10.1021/acsmacrolett.2c00319.
(15) Bannin, T. J.; Kiesewetter, M. K. Poly(Thioester) by Organocatalytic Ring-Opening Polymerization. *Macromolecules* **2015**, *48* (16), 5481–5486. https://doi.org/10.1021/acs.macromol.5b01463.
(16) Wang, F.; Liu, H., Fu, H., Jiang, Y., Yufen, J., Zhao, Y., Highly Efficient Iron(II) Chloride/NBS-Mediated Synthesis of Imides and Acylsulfonamides *Advanced Synthesis and Catalysis* **2009**, *351* (1-2), 246-252. https://doi.org/10.1002/adsc.200800668
(17) Xuan, M.; Lu, C.; Liu, M.; Lin, B.-L. Air-Tolerant Direct Thiol Esterification with Carboxylic Acids Using Hydrosilane via Simple Inorganic Base Catalysis. *J. Org. Chem.* **2019**, *84* (12), 7694–7701. https://doi.org/10.1021/acs.joc.9b00500.
(18) Ogiwara, Y.; Takano, K.; Horikawa, S.; Sakai, N. Indium-Catalyzed Direct Conversion of Lactones into Thiolactones Using a Disilathiane as a Sulfur Source. *Molecules* **2018**, *23* (6), 1339. https://doi.org/10.3390/molecules23061339.
(19) Wang, J.; Wolf, R. M.; Caldwell, J. W.; Kollman, P. A.; Case, D. A. Development and Testing of a General Amber Force Field. *Journal of Computational Chemistry* **2004**, *25* (9), 1157–1174. https://doi.org/10.1002/jcc.20035.
(20) Hanwell, M. D.; Curtis, D. E.; Lonie, D. C.; Vandermeersch, T.; Zurek, E.; Hutchison, G. R. Avogadro: An Advanced Semantic Chemical Editor, Visualization, and Analysis Platform. *Journal of Cheminformatics* **2012**, *4* (1), 17. https://doi.org/10.1186/1758-2946-4-17.
(21) Turney, J. M.; Simmonett, A. C.; Parrish, R. M.; Hohenstein, E. G.; Evangelista, F. A.; Fermann, J. T.; Mintz, B. J.; Burns, L. A.; Wilke, J. J.; Abrams, M. L.; Russ, N. J.; Leininger, M. L.; Janssen, C. L.; Seidl, E. T.; Allen, W. D.; Schaefer, H. F.; King, R. A.; Valeev, E. F.; Sherrill, C. D.; Crawford, T. D. Psi4: An Open-Source Ab Initio Electronic Structure Program. *WIREs Computational Molecular Science* **2012**, *2* (4), 556–565. https://doi.org/10.1002/wcms.93.
(22) Humphrey, W.; Dalke, A.; Schulten, K. VMD: Visual Molecular Dynamics. *Journal of Molecular Graphics* **1996**, *14* (1), 33–38. https://doi.org/10.1016/0263-7855(96)00018-5.
